# DNA-methylome-assisted classification of patients with poor prognostic subventricular zone associated *IDH*-wildtype glioblastoma

**DOI:** 10.1007/s00401-022-02443-2

**Published:** 2022-06-04

**Authors:** Sebastian Adeberg, Maximilian Knoll, Christian Koelsche, Denise Bernhardt, Daniel Schrimpf, Felix Sahm, Laila König, Semi Ben Harrabi, Juliane Hörner-Rieber, Vivek Verma, Melanie Bewerunge-Hudler, Andreas Unterberg, Dominik Sturm, Christine Jungk, Christel Herold-Mende, Wolfgang Wick, Andreas von Deimling, Juergen Debus, Stefan Rieken, Amir Abdollahi

**Affiliations:** 1 German Cancer Consortium (DKTK), Core Center Heidelberg, Heidelberg, Germany; 2 National Center for Tumor Diseases (NCT), Heidelberg University Hospital (UKHD) and German Cancer Research Center (DKFZ), Heidelberg, Germany; 3 Department of Radiation Oncology, Heidelberg University Hospital (UKHD), Im Neuenheimer Feld 400, 69120 Heidelberg, Germany; 4 Heidelberg Institute for Radiation Oncology (HIRO), National Center for Radiation Research in Oncology (NCRO), UKHD and DKFZ, Im Neuenheimer Feld 400, 69120 Heidelberg, Germany; 5 Clinical Cooperation Unit Radiation Oncology, German Cancer Research Center (DKFZ), Im Neuenheimer Feld 280, 69120 Heidelberg, Germany; 6 Clinical Cooperation Unit Translational Radiation Oncology, German Cancer Research Center (DKFZ), Im Neuenheimer Feld 460, 69120 Heidelberg, Germany; 7 Department of Neuropathology, University Hospital of Heidelberg, Im Neuenheimer Feld 224, 69120 Heidelberg, Germany; 8 Clinical Cooperation Unit Neuropathology, German Cancer Consortium (DKTK), German Cancer Research Center (DKFZ), Im Neuenheimer Feld 280, 69120 Heidelberg, Germany; 9 Department of Radiation Oncology, TUM, Ismaninger Str. 22, 81675 Munich, Germany; 10 Division of Pediatric Glioma Research, German Cancer Research Center (DKFZ), Im Neuenheimer Feld 280, 69120 Heidelberg, Germany; 11 Department of Radiation Oncology, University of Texas M.D. Anderson Cancer Center Houston, Houston, TX USA; 12 Genomics and Proteomics Core Facility, German Cancer Research Center (DKFZ), Im Neuenheimer Feld 580, 69120 Heidelberg, Germany; 13 Department of Neurosurgery, University Hospital of Heidelberg, Im Neuenheimer Feld 400, 69120 Heidelberg, Germany; 14 Division of Experimental Neurosurgery, Department of Neurosurgery, University Hospital of Heidelberg, Im Neuenheimer Feld 400, 69120 Heidelberg, Germany; 15 Department of Pediatric Oncology, Hematology, Immunology and Pulmonology, Angelika Lautenschläger Children’s Hospital, University Medical Center for Children and Adolescents, Im Neuenheimer Feld 430, 69120 Heidelberg, Germany; 16 Department of Neurooncology, University Hospital Heidelberg, Im Neuenheimer Feld 400, 69120 Heidelberg, Germany

## Abstract

Glioblastoma (GBM) derived from the “stem cell” rich subventricular zone (SVZ) may constitute a therapy-refractory subgroup of tumors associated with poor prognosis. Risk stratification for these cases is necessary but is curtailed by error prone imaging-based evaluation. Therefore, we aimed to establish a robust DNA methylome-based classification of SVZ GBM and subsequently decipher underlying molecular characteristics. MRI assessment of SVZ association was performed in a retrospective training set of IDH-wildtype GBM patients (*n* = 54) uniformly treated with postoperative chemoradiotherapy. DNA isolated from FFPE samples was subject to methylome and copy number variation (CNV) analysis using Illumina Platform and cnAnalysis450k package. Deep next-generation sequencing (NGS) of a panel of 130 GBM-related genes was conducted (Agilent SureSelect/Illumina). Methylome, transcriptome, CNV, MRI, and mutational profiles of SVZ GBM were further evaluated in a confirmatory cohort of 132 patients (TCGA/TCIA). A 15 CpG SVZ methylation signature (SVZM) was discovered based on clustering and random forest analysis. One third of CpG in the SVZM were associated with *MAB21L2*/*LRBA*. There was a 14.8% (*n* = 8) discordance between SVZM vs. MRI classification. Re-analysis of these patients favored SVZM classification with a hazard ratio (HR) for OS of 2.48 [95% CI 1.35–4.58], *p* = 0.004 vs. 1.83 [1.0–3.35], *p* = 0.049 for MRI classification. In the validation cohort, consensus MRI based assignment was achieved in 62% of patients with an intraclass correlation (ICC) of 0.51 and non-significant HR for OS (2.03 [0.81–5.09], *p* = 0.133). In contrast, SVZM identified two prognostically distinct subgroups (HR 3.08 [1.24–7.66], *p* = 0.016). CNV alterations revealed loss of chromosome 10 in SVZM– and gains on chromosome 19 in SVZM– tumors. SVZM– tumors were also enriched for differentially mutated genes (*p* < 0.001). In summary, SVZM classification provides a novel means for stratifying GBM patients with poor prognosis and deciphering molecular mechanisms governing aggressive tumor phenotypes.

**Supplementary Information:**

The online version contains supplementary material available at 10.1007/s00401-022-02443-2.

## Introduction

Glioblastoma (GBM), the most common primary brain tumor, is characterized by an infiltrative growth pattern and inherent refractoriness to therapy. This inevitably leads to local therapy failure and dismal prognosis, with an overall survival (OS) of approximately 15 months after surgery and chemoradiotherapy [16], and up to 20.5 months with the addition of tumor-treating fields (TTFields) [46]. The resistance of GBM to therapy has been attributed to a variety of factors, such as inter- as well as intra-tumoral heterogeneity, tumor origin, stem cell-like characteristics and tumor-stroma communication at the tumor vessel/immune response [1, 16, 19]. Predictive biomarkers such as promoter methylation status of the DNA repair enzyme O^6^-methylguanine DNA methyltransferase (*MGMT*) was causally linked to enhanced vulnerability of GBM cells to DNA damage inducing agents such as temozolomide chemotherapy [20, 53]. Hence, identification of molecular subtypes has advanced patient selection for ongoing prospective trials, such as the NCT Neuro Master Match (N2M2), a basket trial wherein poor prognostic *MGMT* hypomethylated tumors are molecularly stratified to different targeted therapies administered concurrently with radiotherapy [52].

A growing body of data indicates a prognostic impact for GBM localized to the subventricular zone (SVZ), a 3–5 mm-thick region located adjacent to the lateral ventricles that harbors neural stem cells (NSCs) throughout adult life [4, 5, 24, 25, 36]. These data examined the hypothesis that tumors originating from this stem cell-rich niche may conserve NSC characteristics. Indeed, genetic models have impressively demonstrated that the introduction of a few aberrations selectively targeted to a few hundred NSCs present in the murine SVZ region are sufficient to form tumors with human GBM characteristics [28, 31]. Moreover, ablation of this low-cycling cell population in established tumors was associated with improved prognosis and outcomes [57]. Accordingly, the association of GBM in SVZ areas was attributed to a stem cell-like phenotype with aggressive clinical behavior and poor survival [3, 4]. Classification of SVZ GBM in clinical studies largely relies on non-invasive magnetic resonance imaging (MRI). High interobserver variability and a lack of objective criteria for defining “central” tumors originating from the SVZ region versus secondary infiltration of “peripheral” tumors to this region limits implementation of a robust imaging-based clinical classifier. Accordingly, part of the poor prognosis in MRI-based SVZ GBM may be related to selection for a more invasive and migratory GBM subtype secondarily infiltrating this area [5]. As such, GBM in contact with the SVZ defined by MRI are more likely to recur as multifocal disease [5, 30]. Therefore, reliable molecular classifiers of SVZ GBM are urgently needed [44] to assist physicians in efforts to circumvent the uncertainty of current MRI-based methods.

Pioneering studies have led to introduction of DNA methylome analysis into the clinical realm, revolutionizing tumor classification in neuropathology [9, 13, 14]. A particular strength of this method relates to the long-term preservation of the epigenetic fingerprint, as opposed to more dynamic transcriptome signatures, thereby better allowing for determination of the cell of origin. This study reports on the utilization of DNA methylome analysis for discovery of a novel molecular SVZ classification principle that could potentially improve current imaging-based stratification. The SVZ methylome (SVZM) classifier was subsequently employed for molecular characterization of these tumors on multiple chromosomal aberrations (CNV), mutational signatures (exome), and transcriptome levels.

## Materials and methods

### Patients and tissue sampling.

This study was approved by the Institutional Review Board and Ethics Committee (No. S-056/2015) and retrospectively evaluated 54 isocitrate dehydrogenase-1 (*IDH1*) wild-type GBMs with available tissue samples that completed a course of radiotherapy (RT) in the Department of Radiation Oncology at University Hospital Heidelberg from 2005–2013. Of these patients, radiographic assessment using magnetic resonance imaging (MRI) was conducted for stratification into two cohorts (SVZ– and SVZ+). The latter (also labeled as “central” GBMs) was defined by the contrast-enhanced lesion having infiltrated the borders of the lateral ventricle and SVZ (5 mm margin lateral to the lateral ventricles), which could be accompanied by subependymal spreading (tumor-related contrast enhancement spreading along the ventricle walls). The remainders were categorized as SVZ–, or “peripheral” GBMs. The SVZ– patients were matched with the SVZ+ patients according to tumor localization, performance status, age, availability of sufficient pre-radiotherapeutic tumor tissue samples, and follow-up data.

Of note, because these patients were treated as early as 2005, not all patients received concurrent and adjuvant temozolomide (TMZ), and a variety of RT dose/fractionation regimens were utilized. RT in all patients was delivered as three-dimensional conformal RT. Target volume delineation was based on T1-weighted MRI, and included the primary tumor region, resection cavity (if applicable), and T2-hyperintense areas. A clinical target volume (CTV) margin of up to 2 cm was added on the gross tumor volume (GTV) and resection cavity to account for microscopic tumor spread, respecting anatomic borders. The planning target volume (PTV) was made from the CTV+ a 5 mm margin for setup inaccuracies.

Tumor samples, obtained before commencing RT, were archived in the Department of Neuropathology at the University Hospital of Heidelberg’s Institute of Pathology. As part of contemporary diagnostic assessment based on molecular factors, all tissue samples were re-evaluated based on histopathological criteria, immunohistochemical staining, and molecular analyses according to the 2016 World Health Organization classification of central nervous system tumors [33]. O^6^-methylguanine DNA methyltransferase (*MGMT*) promoter status was determined as previously described [10].

### DNA extraction and molecular analysis

Tumor DNA was extracted from formalin-fixed, paraffin-embedded (FFPE) material. Representative tumor areas with high tumor content (> 80%) were utilized. DNA extraction was performed using the automated Maxwell system (Promega, Madison, WI, USA).

DNA methylation analysis was carried out in the Genomics and Proteomics Core Facility at the German Cancer Research Center (DKFZ, Heidelberg) according to the manufacturer’s instructions as previously described [21]. Genomic positions refer to the hg19 assembly.

GBMs have previously been reported to cluster in distinct subgroups: RTKI, RTKII, *H3.3* K27, and *H3.3* G34 mutated, *IDH* mutated, and mesenchymal (Suppl. Figure 2A and B, online resource) [13]. We additionally selected representative reference cases of each subgroup (*n* = 10 RTK1, *n* = 11 RTKII, *n* = 15 *H3.3* G34 mut., *n* = 19 *H3.3* K27 mut., *n* = 11 *IDH* mut., *n* = 16 mesenchymal) and compared DNA methylation patterns between SVZ – and SVZ + GBMs via unsupervised hierarchical clustering analyses of 30,000 probes showing the highest median absolute deviation (MAD) in beta values (Suppl. Figure 2A, online resource).

CNV data were extracted from methylation arrays utilizing the minfi package. Downstream processing (identification of segments, and automatic threshold selection for the identification of gains/losses) was performed with the cnAnalysis450k package, using references for normal tissue as described earlier [27].

For methylome analysis, idat files were processed with the minfi R package [8, 18]. Data were filtered for probes mapping to chromosome X and Y, as well as single nucleotide polymorphisms (SNPs) and repetitive sequences. Data were funnorm normalized, and M-values were used for analysis. Identification of differentially methylated CpGs between SVZ+ and SVZ– tumors is outlined in Suppl. Figure 1, online resource.

Lastly, for DNA sequencing, the customized SureSelect kit (Agilent), encompassing 130 brain tumor relevant genes, was used for target-enrichment and sequenced on a NextSeq 500 machine (Illumina) as previously described [40]. Mutational data were obtainable for 45 patients, but the DNA quantity was insufficient for the remaining samples.

### Validation dataset

To validate the findings herein using an independent dataset, the GBM data collection (TCGA-GBM) from The Cancer Genome Atlas (TCGA) was utilized. Retrieved data from the TCGA data portal included level 2 Illumina 450 k methylation data, RNASeq data, whole exome sequencing data (level 3), and clinical data (follow_up_v1.0). RNASeq was rlog transformed prior to analysis. Silent mutations were filtered. Enrichment tests between groups were performed using Barnard’s tests per gene and any type of mutation vs no/silent mutation. *IDH1* mutant samples (WXS data) were excluded from analyses. Matching MRI data for the TCGA-450 k cohort were retrieved from The Cancer Imaging Archive (TCIA) and classified as SVZ+ or SVZ– by two independent raters. Patient characteristics of the respective cohorts are shown in Suppl.-Tbl. 1, online resource.

### Survival analysis

To examine the association of SVZ apposition (and correlation with molecular/genetic factors) with clinical outcomes, evaluation of survival was conducted. As a prerequisite to this analysis, all patients were followed up clinically and with MRI 4 weeks after RT, followed by 3 month intervals until recurrence/progression or death. Tumor localization and progression were assessed by an experienced radiologist based on the RANO criteria [51]. Overall survival (OS) was calculated using the Kaplan–Meier method as the time between the date of commencing RT until the date of death, or censored at last contact.

### Statistical analyses

Statistics were carried out in R v3.6.1 R Core Team, [47] and utilized a two-sided α of 0.05 unless stated otherwise. For categorical tests, the chi-squared, Fisher’s, or Barnard tests were used. Survival was additionally assessed using Cox proportional hazard models or parametric survival regressions with the survival package [48]. Random forest analyses were conducted with the randomForest package [29]. The psych and irr packages were used to calculate intraclass correlations. Pathway activity estimation from expression data was performed with PROGENy [43].

## Results

### SVZ classification based on MRI

A retrospective cohort of 54 GBM patients classifiable by MRI for SVZ association were denoted as the training set. Among them, tumors of 30 (55.6%) patients were classified as SVZ– and 24 (44.4%) were SVZ + GBMs as discerned by MRI features. Table 1 demonstrates clinical characteristics of both cohorts.

**Table 1:** Patient characteristics

Patient characteristics [MRI classification]	SVZ+, *n* = 24 (%)	SVZ–, *n* = 30 (%)	*p* value
Gender			0.43
Male	17 (70.8)	17 (56.7)	
Female	7 (29.2)	13 (43.3)	
Age at start RT, year	57.9 [39–81]	59.7 [39–81]	0.78
Karnofsky Performance Status			0.54
> = 80	14	21	
< 80	10	9	
RT dose [Gy]	60 [40.1–60]	60 [45–60]	0.27
Temozolomide			0.33
Yes	14 (58.3)	22 (73.3)	
No	9 (37.5)	8 (26.7)	
Unsure	1 (4.2)	0 (0)	
Surgery			0.08
Subtotal resection	16 (66.7)	11 (36.7)	
Gross total resection	6 (25.0)	16 (53.3)	
Biopsy	2 (8.3)	3 (10.0)	
MGMT promoter			0.53
Hypermethylated	11 (45.8)	10 (33.3)	
Hypomethylated	12 (50.0)	17 (56.7)	
Unsure	1 (4.2)	3 (10.0)	

Chi-squared test for categorical data, *t* test for continuous data [median, range]

*SVZ *subventricular zone, *TMZ* Temozolomide, *MGMT *O6-methylguanine-DNA methyltransferase, MGMT-STP27 classifier; *TMZ*+ adjuvant + concomitant Temozolomide treatment

**Fig. 1:**
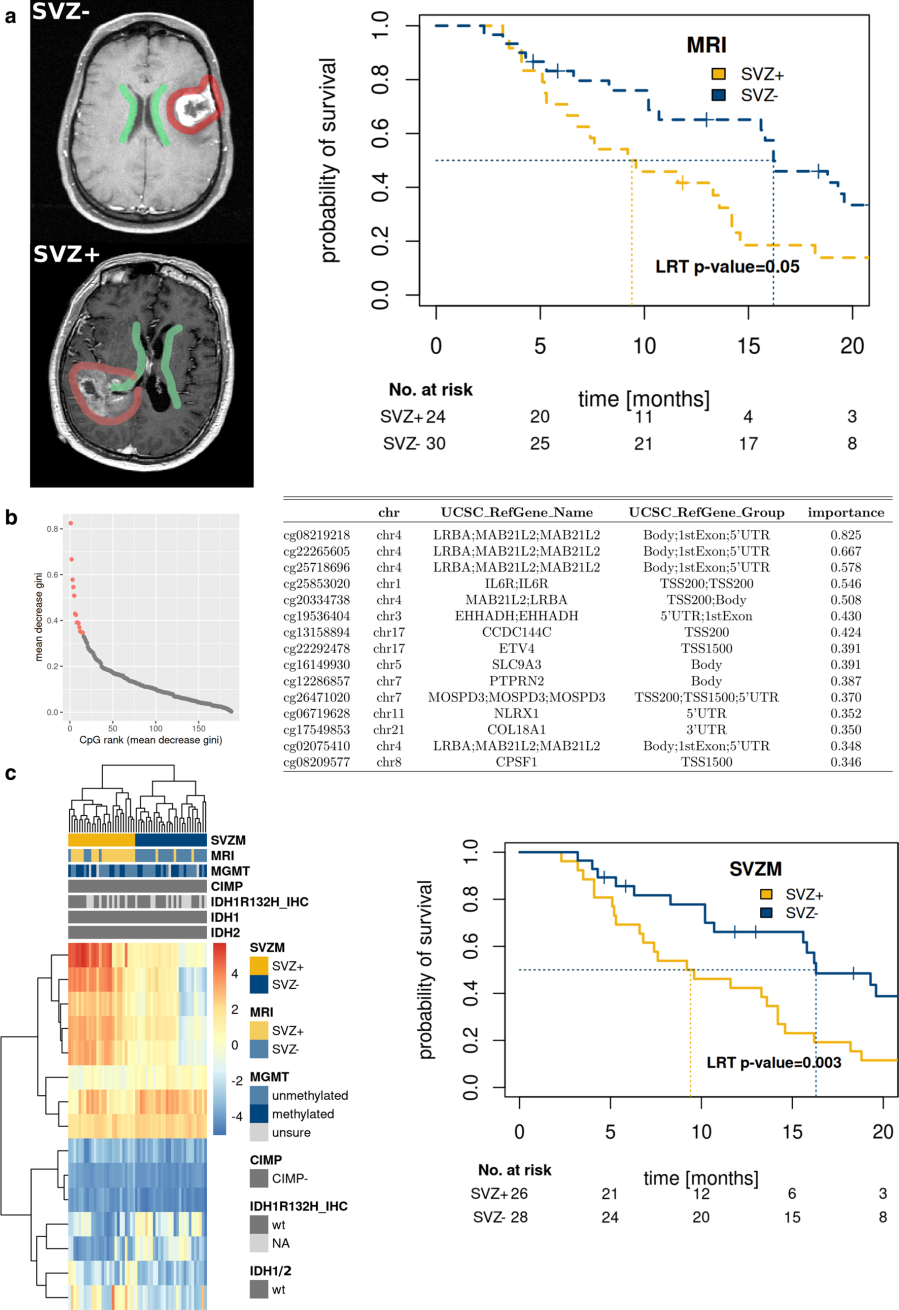
Subventricular zone positive “central” (SVZ+) and subventricular zone negative “peripheral” (SVZ–) glioblastoma differ in their epigenomic signatures. **a** Imaging-based (MRI) classification of GBM patients with poor prognosis SVZ+ tumors (Kaplan–Meier, Cox model likelihood ratio test, LRT). **b** Identification of SVZ specific DNA methylome signature (SVZM) consisting of 15 CpGs. Left: random forest derived rank order of single CpGs according to their relevance to differentiate SVZ state are shown (left red dots, right CpG annotations and importance score). **c** SVZM Classification separates the training cohort into two main clusters (heatmap, hcl with Euclidean distance and complete linkage). Molecular (MGMT, G-CIMP) and MRI classifications are also provided. An inferior prognosis of GBM patients with SVZM+ tumors was found by Kaplan–Meier analysis of patient survival

The vast majority of patients (≥ 90%) underwent resection prior to RT, the median dose of which was 60 Gy (range: 40.1–60). The MRI classified SVZ– group was associated with better OS than the SVZ + patients (*p* = 0.05, LRT, Fig. 1A). All 54 patients were *IDH1/2* wt as determined by panel sequencing.

### Development of a DNA-methylation-based SVZ classifier

The MRI classification was utilized to identify the most relevant differentially methylated probes (DMP, 225 CpGs, defined with *p* < 0.001) using Illumina 450 K microarrays. Hierarchical clustering (HCL) for identification of the least stable classification error between the two modalities and the most relevant DMP among these 188 CpGs were selected by random forest analysis (details in Suppl. Figure 1, online resource). This analysis resulted in a 15 CpG DNA methylation signature distinguishing between SVZ+ and SVZ– tumors (SVZM, Fig. 1B, Suppl, Fig. 11, online resource, upper row). This separation was also seen on global epigenetic level (non-selected CpGs, Suppl. Figure 11, online resource, bottom row). Among the selected CpGs, 5 of the 15 DMP were associated with the *LRBA* gene. Heatmap and HCL of the SVZM classified training cohort combined with other relevant parameters (i.e., MRI evaluation, *IDH1*, CIMP, and *MGMT* status) are provided in Fig. 1C. SVZM clearly separated the training cohort into prognostic subgroups (*p* = 0.003, LRT). No enrichment of SVZ assigned tumors to previously discovered glioma methylation subtypes was found based on hierarchical cluster analysis of most variant probes (Suppl. Figure 2, online resource), using the v11b4 neuropathology classifier [13] revealed that all subtypes were present in SVZM+ and negative tumors (Suppl. Figure 2C, online resource). A previously observed trend between MRI classification and degree of resection (*p* = 0.08, chi-squared test) was weaker for SVZM (*p* = 0.26, Suppl. Figure 3A, online resource). In concordance, Goodman Kruskal’s lambda showed smaller values for SVZM, however the 95% CI included 0 (no association) for all performed comparisons (Suppl. Figure 3A, online resource). Finally, multivariable survival analysis of the extent of resection and SVZ classification method did not show a significant contribution of adding the extent of resection in either case (SVZM: *p* = 0.47 and MRI: *p* = 0.43, Suppl. Figure 3B, online resource).

### Reassessment of misclassified tumors

**Fig. 2:**
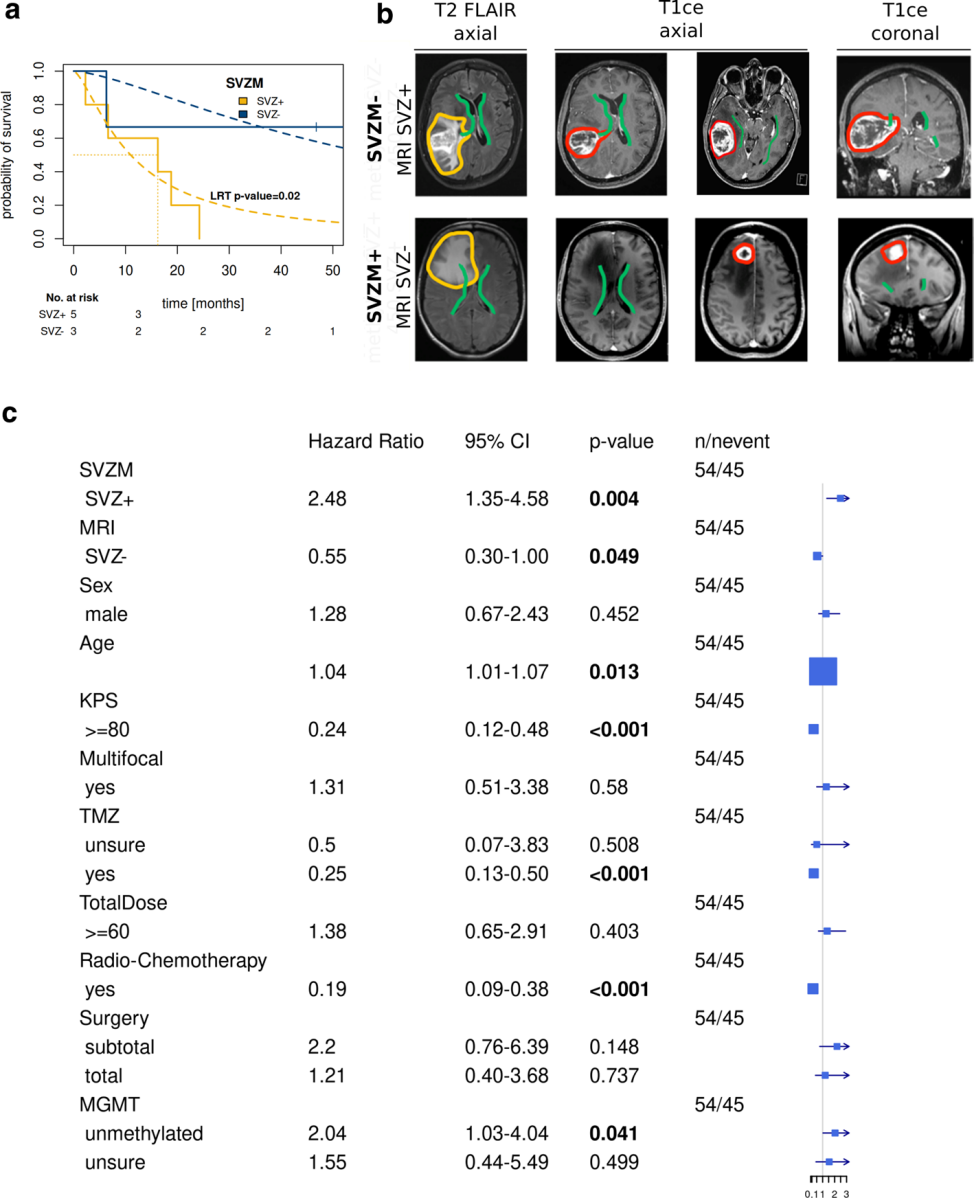
Discordance between SVZM vs. MRI-based classifications. **a** Overall survival of all differently assigned patients indicating an inferior outcome in patients with SVZM+ but according to MRI SVZ negative tumors. Kaplan–Meier curves and parametric survival model (Weibull distribution, dashed line, LRT). **b** Tumor localization of representative patients with discordant classification highlights the difficulty to distinguish between secondary invasion to the SVZ region and tumors originating from this region solely by the imaging method. **c** A significantly increased hazard ratio (HR: 2.48, *p* < 0.004) for SVZM by univariable survival analysis (Cox model) versus other parameter including classifications based on MRI SVZ+ vs SVZ–; female vs male; performance status (KPS ≥ 80 vs < 80); multi- vs unifocal presentation; chemotherapy (TMZ: adjuvant/concurrent vs. incomplete treatment); radiation dose (≥ 60 Gy vs < 60 Gy), surgery: subtotal vs total and MGMT status

As compared to MRI-based classification, eight (14.8%) tumors were reclassified following molecular SVZM assessment. Interestingly, SVZM was able to separate these patients into two distinct prognostic subgroups confirmed on survival analysis (*p* = 0.02 by Weibull distribution, Fig. 2A, updated survival data after extended follow-up). Representative MRI images of tumors with mismatching radiographic and molecular classifications illustrate current difficulties associated with discrimination of SVZ-driven central GBM from secondary infiltration of peripheral tumors into the SVZ. Most MRI SVZ-/SVZM + tumors showed FLAIR signal reaching the SVZ (Fig. 2B, Suppl. Figure 13, online resource). FLAIR signal for classification, however, is only useful if the SVZ region is not reached (Suppl. Figure 14, online resource).

Univariable analysis for OS identified SVZM (hazard ratio HR 2.48, 95% CI [1.35–4.58] *p* = 0.004), MRI SVZ (HR 1.83 [1.00–3.35] *p* = 0.049), age (HR 1.04 [1.01–1.07] *p* = 0.013), performance status (HR 0.24 [0.12–0.48] *p* < 0.001), use of TMZ (HR 0.25 [0.13–0.5] *p* < 0.001), Radio-Chemotherapy (HR 0.19, [0.06–0.38], *p* < 0.001) and *MGMT* promoter methylation status (HR 2.04 [1.03–4.04] *p* = 0.041) as significant covariates (Fig. 2C). Comparative multivariable analysis for MRI-based versus SVZM with clinical (age, performance status) and other molecular/treatment associated covariates (*MGMT* promoter methylation, Radiochemotherapy) showed a significant independent contribution only for the SVZM-based classification with Cox-PH models (Suppl. Figure 4, online resource). After parametric models were applied to compensate for relatively low n, *MGMT* and Radiochemotherapy also showed a statistically significant contribution (Suppl. Figure 12, online resource).

### Validation of the SVZM and molecular characterization

**Fig. 3:**
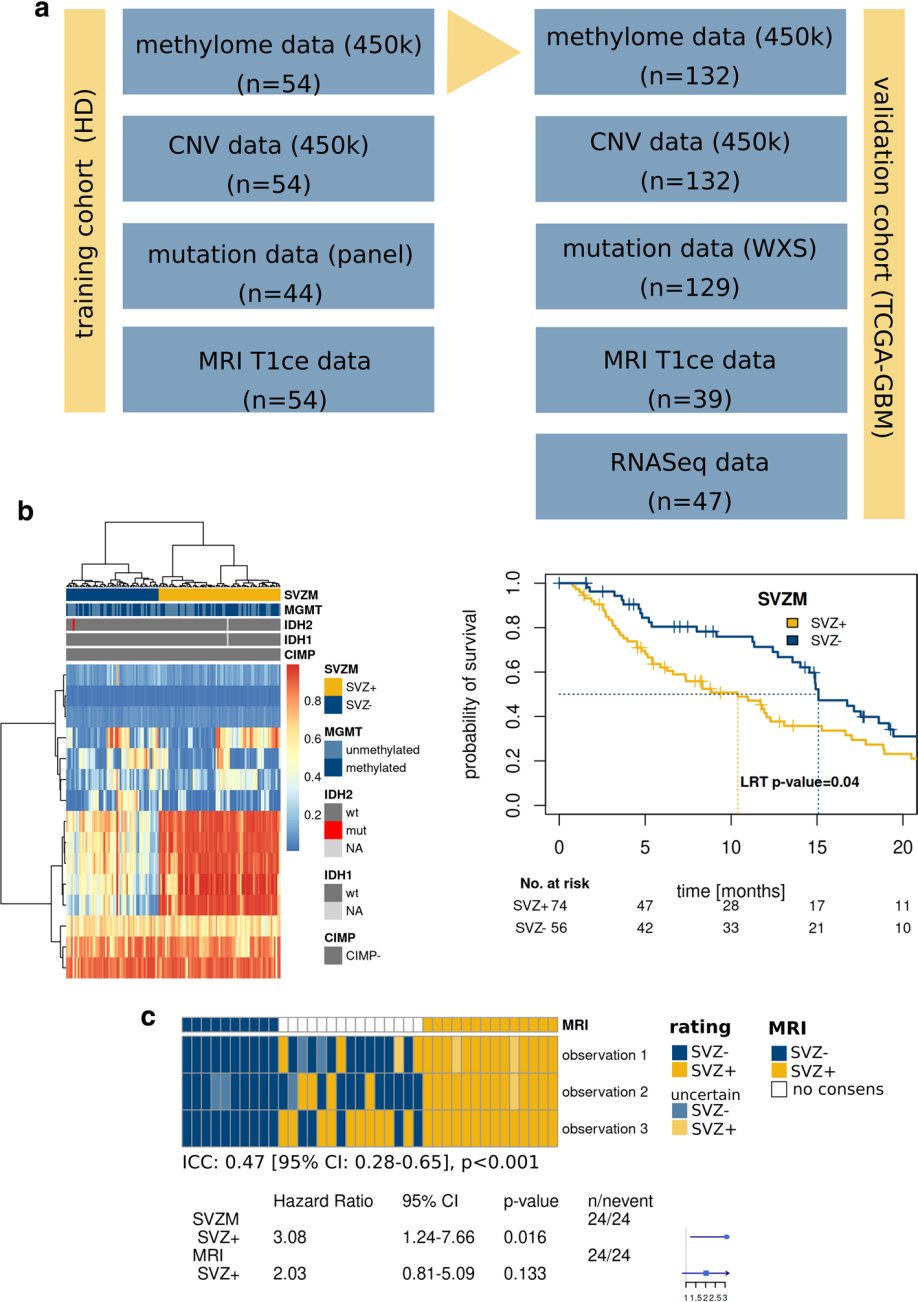
Performance of SVZM vs. MRI in the validation cohort. **a** Overview of multiple layer of data that were correlated with the SVZM state in training/validation cohorts. Methylome and copy number variation (CNV) analysis by 450 K microarrays, T1 contrast enhanced (CE) MRI, mutational profile by whole exome sequencing (WXS), deep “panel” NGS and RNAseq. **b** SVZ± assignment of the validation cohort by cluster analysis of the 15 CpG signature (maximum distance, ward.D) and prognostic evaluation (Kaplan–Meier, Cox model, and LRT). **c** Heterogeneity of SVZ classification by MRI in the validation cohort. Manual rating of patients to SVZ classes based on MRI shows discordance between the three observations for a fraction of patients (intraclass correlation, ICC). Comparative univariable survival analyses (bottom) for the 24 most consistently rated tumors by MRI vs. SVZM (Cox model)

For validation of the SVZM by an independent cohort, TCGA methylome data of 132 GBM patients were utilized. An overview of the available levels of molecular-/imaging data is shown in Fig. 3A. Patients were assigned to SVZM + vs. SVZM– groups via HCL of the 15 DMP of the SVZM signature (Fig. 3B). Two main clusters could be identified, which showed significant differences in OS (*p* = 0.04, LRT). To further validate the proposed SVZM, available matched TCIA MRI imaging data was assessed by a single independent observer yielding an intraclass correlation (ICC) of 0.51 (Fig. 3C). Of note, for 15 out of 39 patients (38%), no consensus classification could be determined by MRI classification. Among the patients with a consensus MRI-based classification, SVZM performed better (SVZM HR 3.08 [CI 1.24–7.66], *p* = 0.016) compared to MRI (HR 2.03 [CI 0.81–5.09], *p* = 0.13) (Fig. 3C).

**Fig. 4:**
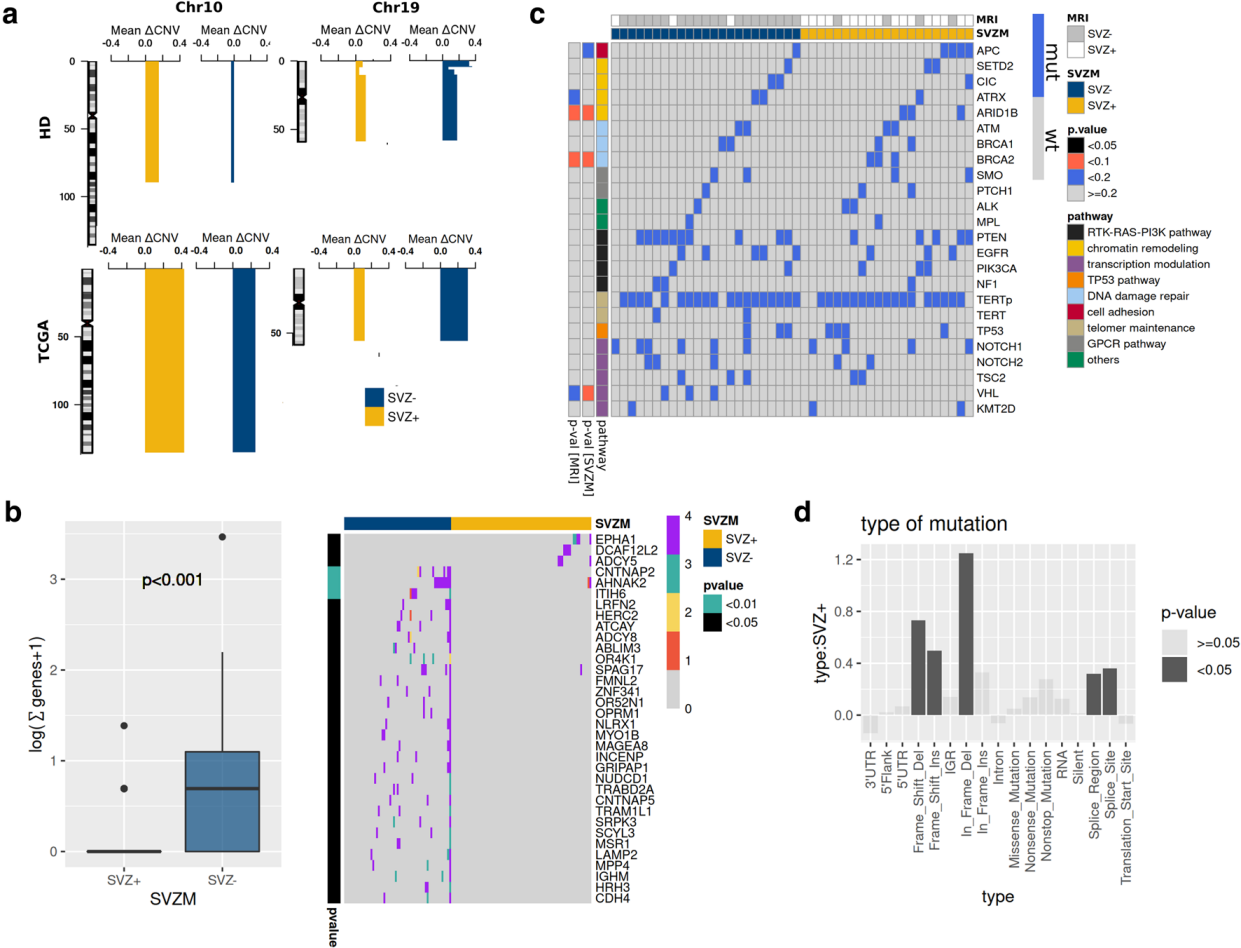
Differential CNV and mutational profile of SVZ GBM. **a** Segmental CNV alterations indicate a relative loss of chromosome 10 in SVZM– and gains on chromosome 19 in SVZM– tumors in both training and validation cohorts. **b** Among differentially mutated genes identified by WXS in the validation cohort, a significantly lower number of mutations in SVZ+ compared to SVZ– tumors was found (left, *p* < 0.001 by Wilcoxon test). Most significantly enriched mutations as a function of SVZ state are shown as heatmap (right, Barnard’s test). Scale bar of the heatmap correspond to non-silent variants, identified as differential between SVZ± in ≥ 3 out of 4 mutation calling pipeline datasets. #calls indicate the number of pipelines identifying a mutation in the respective sample. **c** Differentially enriched mutations as a function of SVZ state identified by ultra-deep panel NGS of the training HD-Cohort. *p* value: Barnard’s test for associations between mutational enrichment in SVZM or SVZ-MRI classified groups, respectively. **d** Interactions between the type of mutation and SVZM status using a linear mixed model (random factor variant calling method) indicate significant association between SVZM+ frameshift (insertion/deletion), in frame deletions and splice region/sites mutations (black bars)

Segmental CNV alterations revealed loss of chromosome 10 in SVZM– tumors and gains on chromosome 19 in SVZM– tumors (Fig. 4A). Segmental and gene/transcript level CNVs are shown in Suppl. Figure 6A and B, online resource. On whole exome sequencing data (WES, TCGA cohort), evaluation of variant calls from four pipelines revealed differentially enriched mutations mostly confined to SVZM– tumors (Fig. 4B), whereas the total number of non-silent mutations did not differ between the two classes (Suppl. Figure 7A, online resource). More specifically, frame shift deletions/insertions, in-frame deletions, mutations in splice regions, and splice sites were enriched in SVZM + tumors (Fig. 4D). *EPHA1*, *DECAF12L2* and *ADCY5* mutations were detected exclusively in SVZM + tumors, whereas *CNTNAP2*, *AHNAK2* and *ITIH6* mutations were among the most significantly enriched in SVZ– tumors (*p* < 0.01) (Fig. 4B). Deep panel sequencing of the Heidelberg cohort revealed no mutational enrichments as a function of SVZM classification; at 10% FDR for the exclusive presence of *ARID1B1* and *BRCA2* in SVZ+, and *VHL* in SVZM– tumors. Cross-comparison of mutational readouts between the Heidelberg and TCGA cohorts was limited by differences in methodology (e.g., sequencing depth and consequently VAF cut-off criteria applied to define mutations). Therefore, it was not surprising that repetitive elements difficult to detect with WES such as *TERTp* mutations were found in 46 (85%) samples of the Heidelberg cohort but not reported in the TCGA study (Fig. 4C). Accordingly, we failed to confirm an exclusive enrichment of mutations in SVZ+ tumors identified in the Heidelberg cohort with TCGA WES data (Suppl. Figure 7C, online resource).

**Fig. 5:**
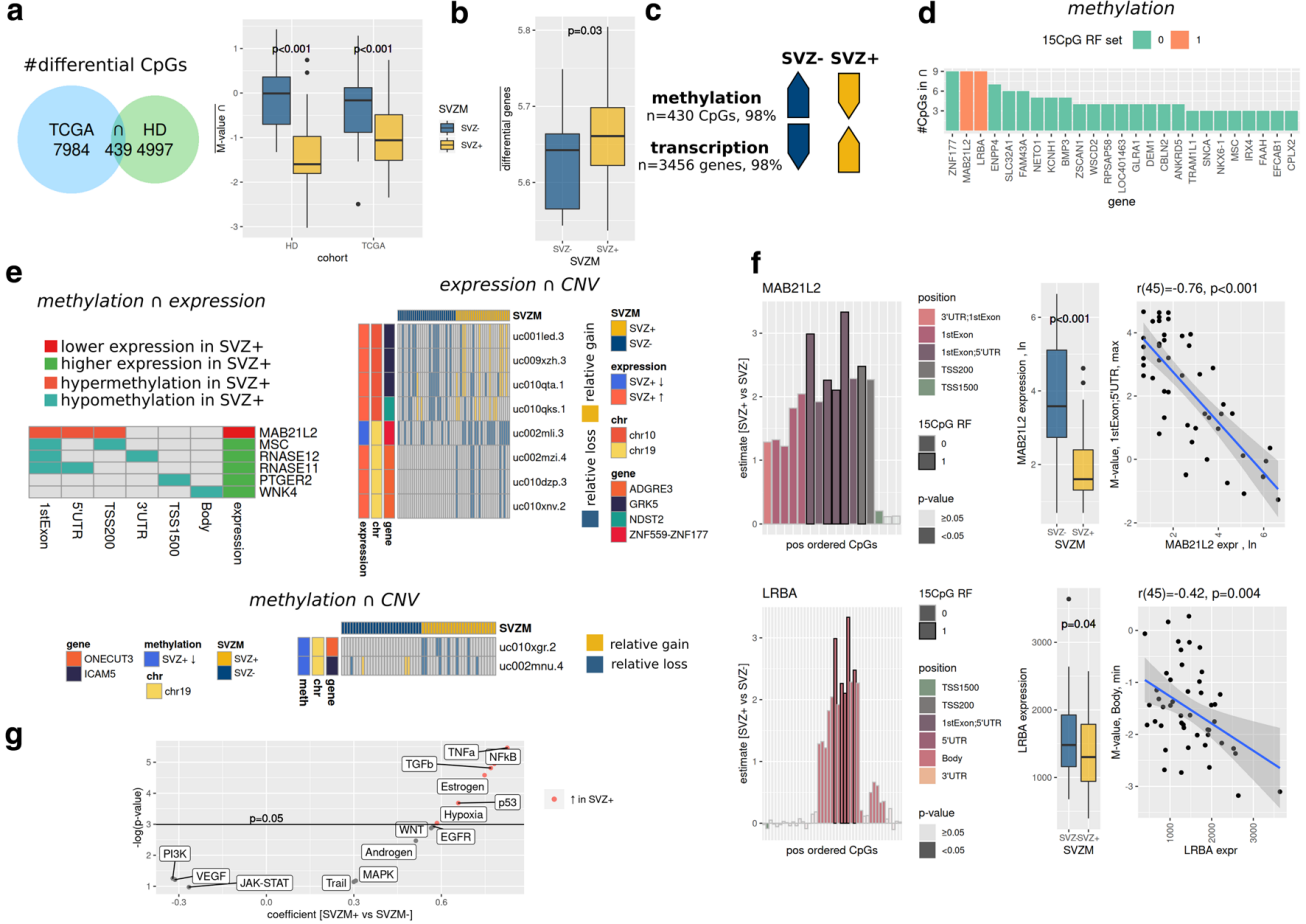
Molecular characterization of SVZ GBM via integrative omics. **a** A consensus set of 439 SVZM associated differentially methylated probes, intersect between training (HD) and validation (TCGA) cohort, was identified. A significant relative hypomethylation in SVZM+ compared to SVZM- tumors was found (right, mean methylation of all CpGs, test: linear model). **b** 3456 genes showed an inverse gene expression vs. CpG methylation pattern. **c** In line with CpG hypomethylation pattern, an enhanced mean gene-expression was found in SVZM+ tumors (selection: t-test, FDR < 0.05. test: linear model). **d** Rank ordered genes based on the number of differentially methylated CpG sites found in the 439-consensus signature. Of note, MAB21L2/LRBA from the SVZM random forest classifier (15 CpG RF set) are with 9 CpGs among the top ranked genes. **e** Intersection between different molecular layers showing an inverse relationship between methylation and expression for MAB21L2 (less expressed in SVZ+) and additional genes higher expressed. Expression and high-resolution CNV alterations show overlap for GRK5, NDST2, ZNF559-ZNF117, and ADGRE3. Methylation and CNV show hypomethylation and relative loss for ICAM5 and ONECUT3 (CNV: *p* < 0.05, Fisher test; methylation: FDR < 0.05, SAM; expression: FDR < 0.05, *t* test). **f** LRBA and MAB21L2 methylation (left, *n* = 132, linear model), expression (middle, *n* = 47, neg-binomial model, count data, log offset [total counts], one-sided p value, transformation for visualization) and correlation between methylation and expression (*n* = 47, Pearson). **g** PROGENy inferred enhanced pathway activity in SVZM+ tumors derived from RNAseq data, linear model analysis

A consensus set of 439 CpGs was identified as being differentially methylated in both cohorts as a function of SVZM status (FDR < 0.05 by SAM with 500 permutations, Fig. 5A and Suppl. Figure 5A, online resource). DMPs were significantly hypomethylated in SVZM + vs. SVZM– tumors in both studied cohorts (p < 0.001). Global alterations on methylome and gene expression levels revealed an inverse relationship between DMPs and differentially expressed genes (DEG). The mean expression of DEGs was significantly increased in SVZ + tumors (Fig. 5B), showing a hypomethylation of 430 CpGs and higher expression of 3456 genes (total regulated genes: 3456 + 55, *n* = 55 being in average less expressed in SVZM + tumors, *t* test, FDR < 0.05 for selection of differential genes, Fig. 5C).

With 9 CpGs, *MAB21L2*, *LRBA*, and *ZNF177* were the most frequently abundant in the consensus 439 SVZ associated DMPs (Fig. 5D, Suppl. Figure 5C, online resource). The *LRBA* and *MAB21L2* CpGs are located on chromosome 4 on overlapping positions, and *ZNF177* is located on chromosome 19 (Suppl. Figure 5C+D, online resource). A paralog of *ZNF177* on chromosome 19 (hypomethylated in SVZ +), *ZNF559-ZNF177*, showed a CN loss in SVZ+ and was less expressed, whereas *ZNF177* did not show a significant difference in expression (Suppl. Figure 8, online resource). Five of the 9 *LRBA*/*MAB21L2* annotated and differentially methylated CpGs were part of the SVZM signature (Suppl. Figure 5B, online resource). The next highest ranked (≥ 6CpGs) were *ENPP4*, *SLC32A1* and *FAM43A*. *NETO1*, *KCNH1* and *BMP3* were present with > 3 CpGs. Additional overlaps between all DMPs and 15CpG RF signature were detected for *CPSF1* and *EHHADH* (Suppl. Figure 5B, online resource).

Pair-wise co-alterations of SVZM differential genes (methylation [M], expression [E], copy number [CN]) are shown in Fig. 5E. In SVZ+ , co-regulation analysis between expression and methylation identified *MAB21L2* (high E/low M), and for low E/high M *MSC*, *RNASE11*, *RNASE12*, *PTGER2* and *WNK4*. Expression vs copy number analysis identified *GRK5* and *NDST2* with high E/CN gain, *ZNF599-ZNF177* low E/CN loss, and *ADGRE3* high E/CN loss. Methylation and copy number co-alteration showed low M/CN loss for *ONECUT3* and *ICAM5*.

SVZ± population-based testing for differences in *LRBA* and *MAB21L2* expression revealed decreased expression in SVZ+ for both genes. Moreover, a negative correlation between methylation pattern and gene-expression was found (Fig. 5F, – 0.76 for *MAB21L2*, -0.42 for *LRBA*, Pearson *p* < 0.01). Detailed methylation analysis of *MAB21L2* and *LRBA* showed significant hypermethylation in 14 out of 16 *MAB21L2* annotated CpGs (88%), and 19 CpGs located in the body region of *LRBA* (Fig. 5F, Suppl. Figure 5C, online resource). Potential sources of *LRBA* might be T-cells and/or microglia (Suppl. Figure 10 [50]), a direct link to tumor cells seems less likely (Suppl. Figure 9 [38, 56]).

Finally, we performed more global pathway activity estimation from expression data which revealed higher inferred activity for TNFα, NFκB, TGFβ, estrogen, p53, and hypoxia in SVZM + tumors (*p* < 0.05, Fig. 5G).

## Discussion

This study reports on the discovery of a novel molecular classifier of SVZ-driven GBM based on DNA methylome analysis – the SVZM. Based on the growing implementation of DNA methylome analysis in neuropathology [13, 14, 24, 55], the existence of such classifiers could be of utmost relevance for designing prospective studies where SVZM complement current MRI-based classification for a more accurate and robust stratification of patients. In the training cohort, where patients with a clear consensus MRI-based SVZ association were selected to guide methylome classifier development, clustering based on the SVZM signature showed superior prognostic performance. Moreover, detailed re-analysis of MRI data based on SVZM assignment in SVZM vs. MRI discordant cases provided a plausible explanation for a possible erroneous MRI classification of peripheral tumors with secondary infiltration to the SVZ region or contact to the SVZ on T2 sequence data. This is also consistent with the improved performance of SVZM on multivariable analysis and its ability to discriminate prognostic subgroups among the discordantly classified patients. In addition to secondary infiltration as a source for the MRI classification error, heterogeneity in treatment and clinical variables as well as known prognostic subgroups such as enrichment for mutant *IDH*/G-CIMP in peripheral tumors might contribute to differential outcomes attributed to SVZ status [25]. Therefore, IDH-mut/G-CIMP positive patients were excluded in our study and clinically relevant parameters were well balanced in our training cohort. Moreover, for detection of the tumor cell of origin, preservation of the epigenetic fingerprint by DNA-methylome analysis may pose advantages over more dynamic molecular readouts such as transcriptome analysis. Together, MRI classification bias with potential enrichment for invasive tumors in the SVZ group (secondary infiltration of peripheral tumors), heterogeneity and unintended enrichment for prognostic subgroups as well as contamination of stroma cell signatures by analysis of tumor bulk might provide plausible explanations for previous failure to molecularly characterize SVZ GBM as a distinct biological subgroup [37]. Consequently, recent attempts to reduce the influence of the aforementioned variables (e.g., by excluding *IDH*mut tumors) reported successful characterization of SVZ GBM as a distinct gene-expression subtype with enrichment of cancer stem cell-like markers (e.g., *CD133*) and increased expression of genes associated with Notch and DNA-repair pathways [24, 45].

Male abnormal 21 (*MAB21*) homolog protein *MAB21L2* (Mab-21 Like 2) and *LRBA* (lipopolysaccharide-LPS–responsive vesicle trafficking, beach- and anchor-containing) build a nested gene pair (embedding of one gene in another), which is a unique evolutionarily conserved feature reaching back to *C. elegans* [49]. MAB21 like protein family members are linked to cell fate determination, neuronal development, and increasingly functionally connected to the immune response. In addition to involvement in key immune pathways such as TGF signaling described for *MAB21L2*, their nucleotidyltransferase activity has been recently studied and compared with another prominent MAB21 family member, *cGAS* (cyclic GMP-AMP synthase, also known as MAB21 domain-containing protein 1–*MB21D1*), which is a pivotal cytosolic DNA sensor and activator of the innate immune system [15]. Intriguingly, 14/16 SVZM+ DMPs and 1/3 of the classifier was dominated by probes related to *MAB21L1*/*LRBA*, all demonstrating a hypermethylation pattern further correlated with decreased expression in SVZM+ tumors. *Mab21L2* expression is considered for classification of medulloblastoma subtypes (is among the Nanostring signature genes [39]), differentially expressed in brain vs. bone metastases of breast cancer [26], and low expression of *lnc-MAB21L2-1* correlated with resistant to neoadjuvant chemoradiotherapy in rectal cancer [17]. Silencing of *MAB21L2*, as a TGFβ transcriptional repressor, was shown to induce an immune-suppressive microenvironment in myelodysplastic syndrome (MDS) [41]. Accordingly, we found increased TGFβ pathway activity in SVZM+ tumors. *MAB21L2* hypermethylation was associated with chemotherapy resistance in gastric cancer [34], could discriminate between different thyroid tumors [35], was among the top hypermethylated DMP in pathology-free regions of multiple sclerosis-affected brains [22], associated with neurogenesis [55], as well as linked to neural differentiation and human hippocampal neurogenesis in Alzheimer’s disease [6]. Additionally, *LRBA* was shown to be involved in trafficking key immune checkpoints (e.g., *CTLA-4*), is known to contribute to immune dysregulation [32], and is correlated with both disease mortality and recurrence in breast cancer [7]. The source cell of *LRBA*, however, remains elusive [50, 56]. Together with the epigenetic and transcriptional silencing of *MAB21L2*/*LRBA* in SVZM+ tumors found in our study, these data provide a plausible explanation for a prognostic and potentially functional relevance of these genes at the tumor immune microenvironment interface contributing to the observed inferior clinical outcome in SVZM+ GBM. This hypothesis is further supported by the presence of probes associated with IL-6 immune signaling and collagen-18, a precursor to the endogenous angiogenesis inhibitor endostatin [2], in the SVZM classifier and warrants further investigation.

Multiscale molecular characterization of SVZM+ tumors further revealed a global hypomethylation (98% of SVZ associated DMP) and increased gene expression in SVZM+ vs. SVZM– tumors as well as segmental CNV alterations and a significant enrichment for differentially mutated genes in SVZ– tumors. Differential regulation patterns as a function of SVZM status was found on two or more levels, such as in the *ZNF599-ZNF177* locus (CNV, methylation and expression). Interestingly, *ARID1B1* and *BRCA2* mutations were found to be SVZM+ tumor exclusive by deep NGS of the Heidelberg cohort as well as *EPHA1*, *DECAF12L2*, and *ADCY5* by WES in the TCGA cohort. Neither *ARID1B1* nor *BRCA2* were detected in the unselected original Heidelberg GBM cohort evaluated for this panel [40], suggesting that MR-based enrichment of the cohort for SVZ GBM was relevant for this discovery. Moreover, our gene panel constituted relevant mutations in adult as well as pediatric neuro-oncology. Therefore, the presence of mutations such as *ARID1B1* in 3/21 (~ 14%) of SVZM+ tumors was a relatively surprising finding. *ARID1B* mutations are known to appear in ~ 10% of neuroblastoma patients and are correlated with poor clinical outcome. *ARID1B* mutations and alterations are also believed to serve as a driver of tumorigenesis in a small fraction of medulloblastoma and other solid tumors such as breast and ovarian cancers [42]. Exclusive association of *BRCA2* mutations in ~ 14% of SVZM+ provide another interesting target for therapeutic targeting such as synthetic lethal interactions with DNA-damage repair inhibitors and radiotherapy as the cornerstone of postoperative therapy. Among the SVZM+ exclusive mutations identified in the TCGA cohort, the ephrin family member *EPHA1* mutation may provide an interesting therapeutic target as it was recently attributed to an improved response to anti-*PD-L1* immune checkpoint inhibition in lung cancer [11]. Furthermore, *DECAF12L2* could be an attractive biomarker for further investigation as it is among the top 15 driver mutations of GBM (Suppl. Figure 7C, online resource).

Unfortunately, we were not able to validate our deep panel NGS finding with TCGA WES data. This might be due to the limited depth of WES and prefiltering criteria leading to selection of genes with VAF > 50% and other technical issues. For example, *TERT* promoter (pTERT) mutations were rarely found in the TCGA analysis; from 291 GBM patients with WES data 42 had whole genome NGS but only 25 samples had adequate coverage (read count > 10) of the *TERT* promoter for mutational analysis [12]. In contrast, by inclusion of intronic/non-coding regions to cover the *TERT* promoter with an average coverage of 550-fold, we found pTERT mutations in 85% (*n* = 46) of the Heidelberg GBM cohort. The depth of reads might also have been advantageous for detection of genes with large exons like *BRCA1* and its association with SVZM+ in our training cohort. These limitations notwithstanding, these findings warrant further validation in well-powered prospective cohorts and may have ramifications for improved diagnostic and therapeutic tailoring of SVZ+ GBM.

## Supplementary Information

Below is the link to the electronic supplementary material.

Supplementary file1 (PDF 15722 kb)

Supplementary file2 (TIFF 611 kb)
