# Supplementary material for: DNA-methylome-assisted classification of patients with poor prognostic subventricular zone associated IDH-wildtype glioblastoma
Source: Acta Neuropathol. 2022 Jun 4;144(1):129–42. doi: 10.1007/s00401-022-02443-2 (PMC9217840; doi:10.1007/s00401-022-02443-2)
Supplement: Supplementary file 1 — Supplementary file1 (PDF 15722 kb) [file 401_2022_2443_MOESM1_ESM.pdf]

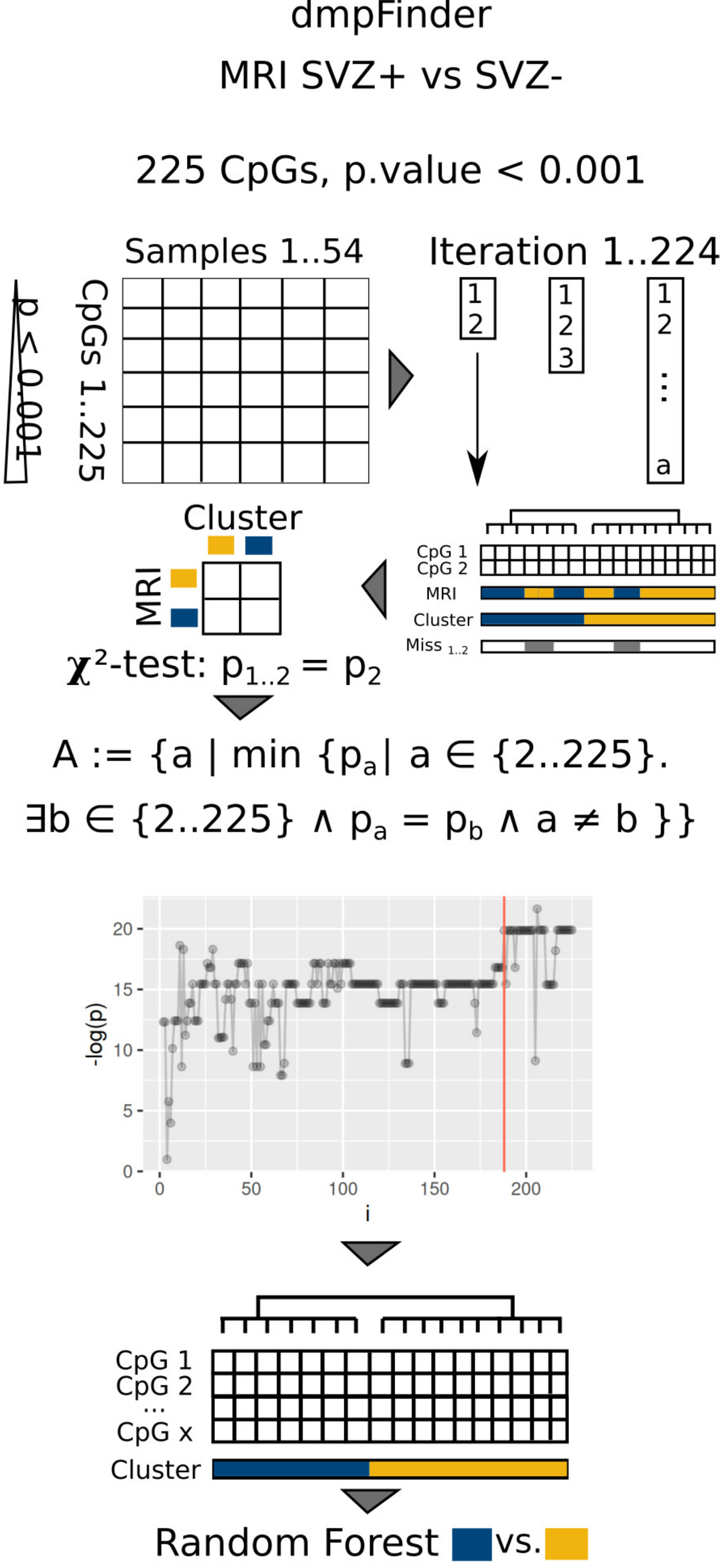

Supplementary Figure 1: Identification of SVZ+/- methylome signature in HD training cohort. For identification of most differentially methylated probes of the 450k methylation array, dmpFinder (minfi package) was utilized and per CpG differences between MRI based SVZ+/- assignments was calculated (no p-value adjustment). Th identify the most relevant CpGs and to adjust for missclassifications, the first up to the nth CpG of the 225 best ranked (p<0.001) CpGs were selected to obtain two main clusters by hierarchical cluster analysis (Euclidean distance, complete linkage). Eahc cluster derived classification was tested against the original MRI based SVZ+/- based assignment and a Chi-Squared test was calculated. The minimum p-value occurign at least twice (CpG 188, orange red vertical line) was used to a obtain a reference clustering on which machine learning random forest classifier was trained.

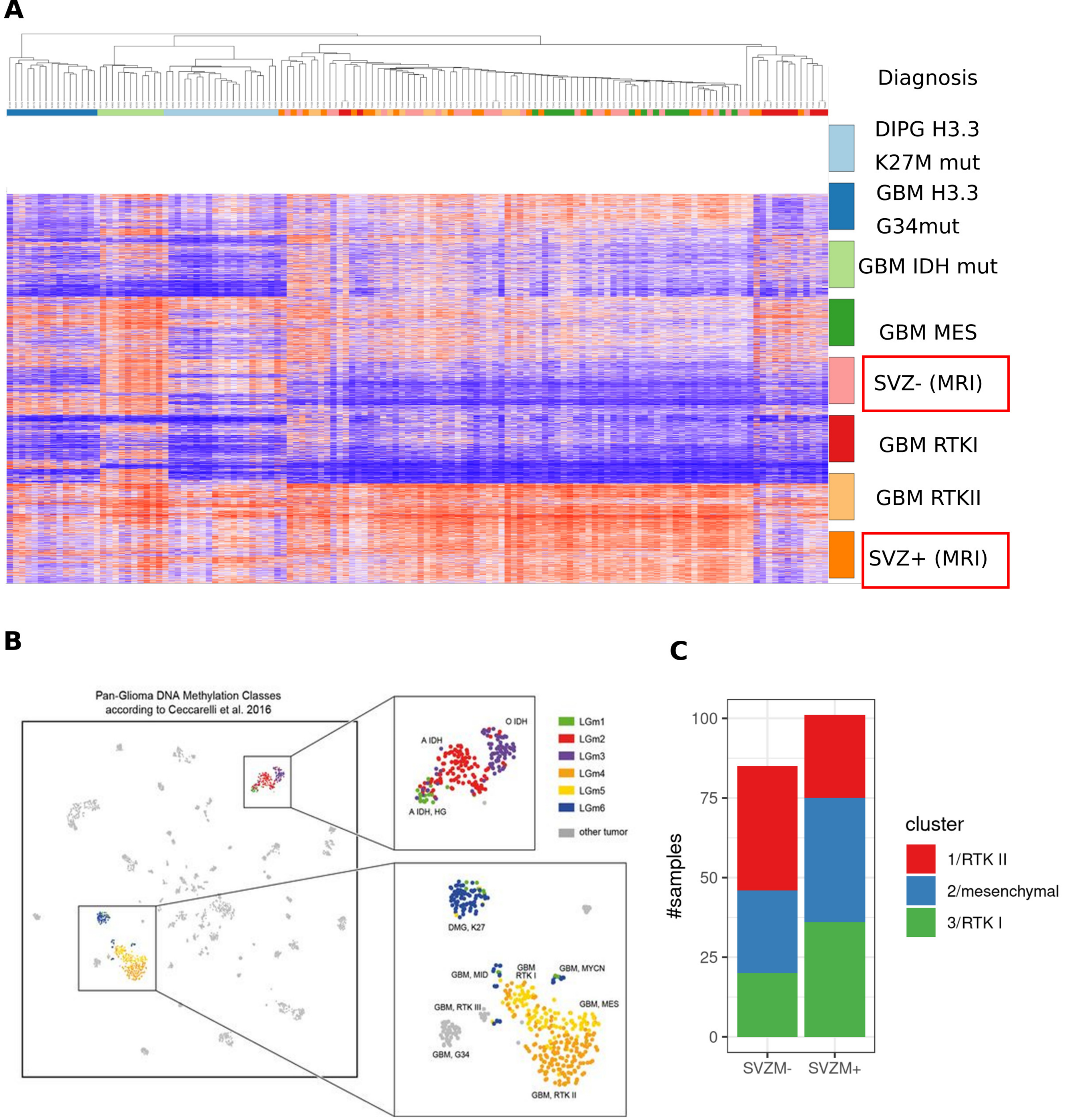

Supplementary Figure 2: Cluster analysis with reference tumors from Heidelberg Neuropathology Department and their corresponding Pan-Glioma DNA-Methylation subtype assignment (RTKI: n=10, RTKII: n=11, H3.3 G34 mut: n=15, H3.3 K27 mut: n=19, IDHmut: n=11 and mesenchymal: n=16) together with the SVZ-HD cohort (n=54) and corresponding MRI SVZ+/- assignments (A). SVZMRI+/- tumors cluster together with GBM-IDH-wildtype subtypes (RTKI/II and MES). There was no apparent further subclustering /enrichment found, unless for RTKI within this main branch. Heatmap shows unsupervised clustering, Euclidean distance and Wards linkage of 30.000 differentially regulated CpGs selected by median absolute deviation (MAD). B: DNA methylation based subtype classification into RTKI/II/MES adapted from [1] shows the relative proximity of these subclasses compared to other glioma or CNS tumor entities. C: Heidelberg DNA-Methylome Classifier (https://www.molecularneuropathology.org/mnp) Version v11b4 assignment of all SVZM positive and negative tumors identified in this study (comined HD and TCGA cohort) shows presence of RTK I/II and MES GBM subtypes independent of SVZM assignment. ComBat was used to merge 450k data from both cohorts and k-means clustering for subtype assignment of TCGA cohort.

[1] Capper, D., et al. DNA methylation-based classification of central nervous system tumours. Nature 555, 469-47 (2018).

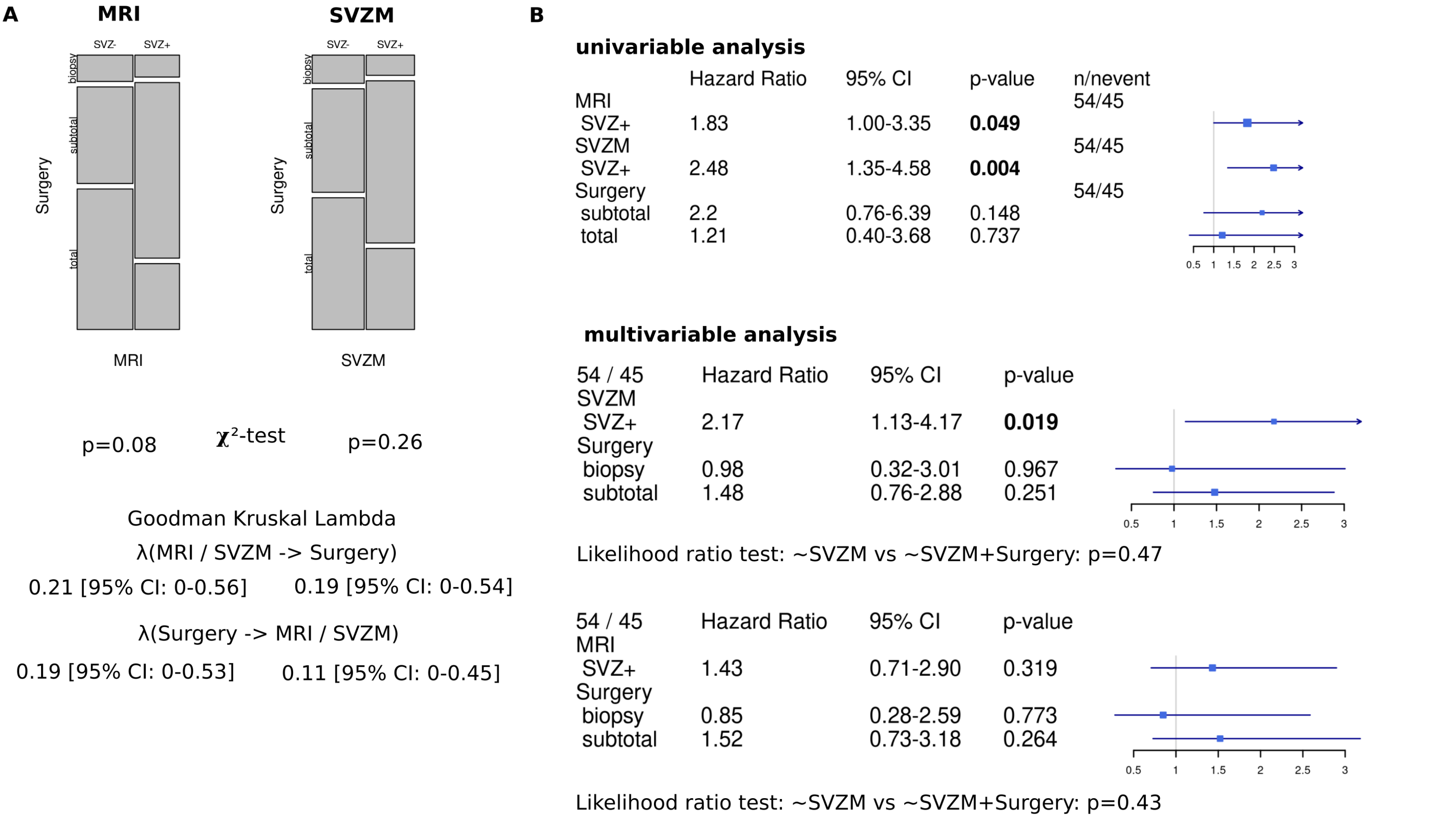

Supplementary Figure 3: Association between degree of resection and SVZ assignment. A: Survival analysis with SVZM/MRI assignment and degree of surgery (reference: gross total resection). Cox models. B: Mosaic plot and measurements of association between both variables.

A

54 / 45	Hazard Ratio	95% CI	p-value
SVZM			
SVZ+	2.5	1.33-4.71	**0.005**
Age			
	1.02	1.00-1.05	0.101
KPS			
>=80	0.3	0.14-0.63	**0.002**

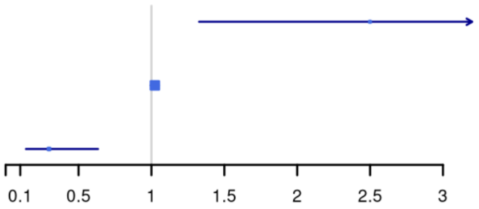

54 / 45	Hazard Ratio	95% CI	p-value
MRI			
SVZ-	0.58	0.31-1.08	0.084
Age			
	1.02	1.00-1.05	0.105
KPS			
>=80	0.3	0.14-0.62	**0.001**

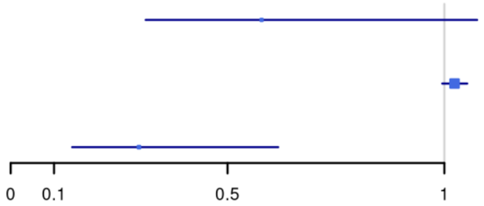

B

50 / 42	Hazard Ratio	95% CI	p-value
MRI			
SVZ-	0.72	0.37-1.38	0.321
Radio-Chemotherapy			
yes	0.2	0.09-0.43	**<0.001**
MGMT			
unmethylated	2.17	1.08-4.34	**0.029**

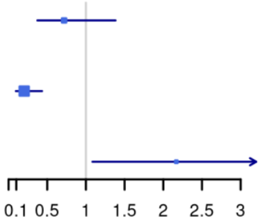

50 / 42	Hazard Ratio	95% CI	p-value
SVZM			
SVZ+	1.98	1.00-3.91	0.05
Radio-Chemotherapy			
yes	0.22	0.10-0.46	**<0.001**
MGMT			
unmethylated	1.86	0.91-3.80	0.089

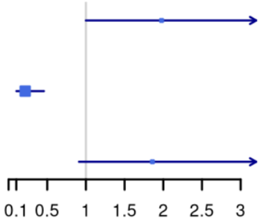

Supplementary Figure 4: Prognostic value of SVZM vs. MRI based SVZ+/- classification in multivariable survival analyses of additional clinical {KPS, Age} (A) molecular/treatment {MGMT, Radio-Chemotherapy} (B) variables (Cox-PH model; SVZ+ vs SVZ-; female vs male; KPS >= 80 vs < 80; unmethylated vs methylated). As *MGMT* methylation unsure classified samples were excluded from analysis.

A

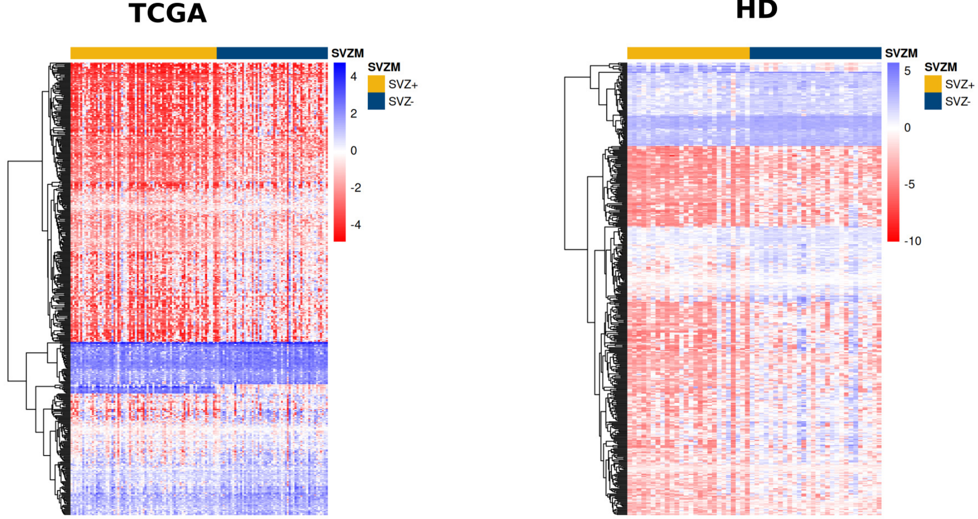

B

	chr	pos	UCSC_RefGene_Name	UCSC_RefGene_Group
cg19536404	chr3	184, 971, 825	EHHADH;EHHADH;EHHADH;EHHADH	5'UTR;1stExon;1stExon;5'UTR
cg20334738	chr4	151, 502, 939	MAB21L2;LRBA	TSS200;Body
cg08219218	chr4	151, 503, 501	LRBA;MAB21L2;MAB21L2	Body;1stExon;5'UTR
cg22265605	chr4	151, 503, 546	LRBA;MAB21L2;MAB21L2	Body;1stExon;5'UTR
cg25718696	chr4	151, 503, 588	LRBA;MAB21L2;MAB21L2	Body;1stExon;5'UTR
cg02075410	chr4	151, 504, 138	LRBA;MAB21L2;MAB21L2	Body;1stExon;5'UTR
cg08209577	chr8	145, 635, 217	CPSF1	TSS1500

C

CpG	Chr	Pos?	UCSC_RefGene_Name	UCSC_RefGene_Group	Signature*
cg05878390	chr4	151, 502, 935	MAB21L2;LRBA	TSS200;Body	
cg20334738	chr4	151, 502, 939	MAB21L2;LRBA	TSS200;Body	1
cg08219218	chr4	151, 503, 501	LRBA;MAB21L2;MAB21L2	Body;1stExon;5'UTR	1
cg22265605	chr4	151, 503, 546	LRBA;MAB21L2;MAB21L2	Body;1stExon;5'UTR	1
cg25718696	chr4	151, 503, 588	LRBA;MAB21L2;MAB21L2	Body;1stExon;5'UTR	1
cg16385330	chr4	151, 503, 878	LRBA;MAB21L2;MAB21L2	Body;1stExon;5'UTR	
cg02075410	chr4	151, 504, 138	LRBA;MAB21L2;MAB21L2	Body;1stExon;5'UTR	1
cg02580005	chr4	151, 504, 187	LRBA;MAB21L2	Body;1stExon	
cg06215107	chr4	151, 504, 725	LRBA;MAB21L2	Body;1stExon	
cg05250458	chr19	9, 473, 565	ZNF177	TSS200	
cg05928342	chr19	9, 473, 598	ZNF177	TSS200	
cg13703871	chr19	9, 473, 674	ZNF177	TSS200	
cg08065231	chr19	9, 473, 684	ZNF177	TSS200	
cg09578475	chr19	9, 473, 688	ZNF177	TSS200	
cg09643544	chr19	9, 473, 696	ZNF177;ZNF177	1stExon;5'UTR	
cg12089570	chr19	9, 473, 715	ZNF177;ZNF177	1stExon;5'UTR	
cg24189904	chr19	9, 473, 781	ZNF177	5'UTR	
cg17283453	chr19	9, 473, 880	ZNF177	5'UTR	

? hg19
* Part of the SVZ+/- methylome signature

D

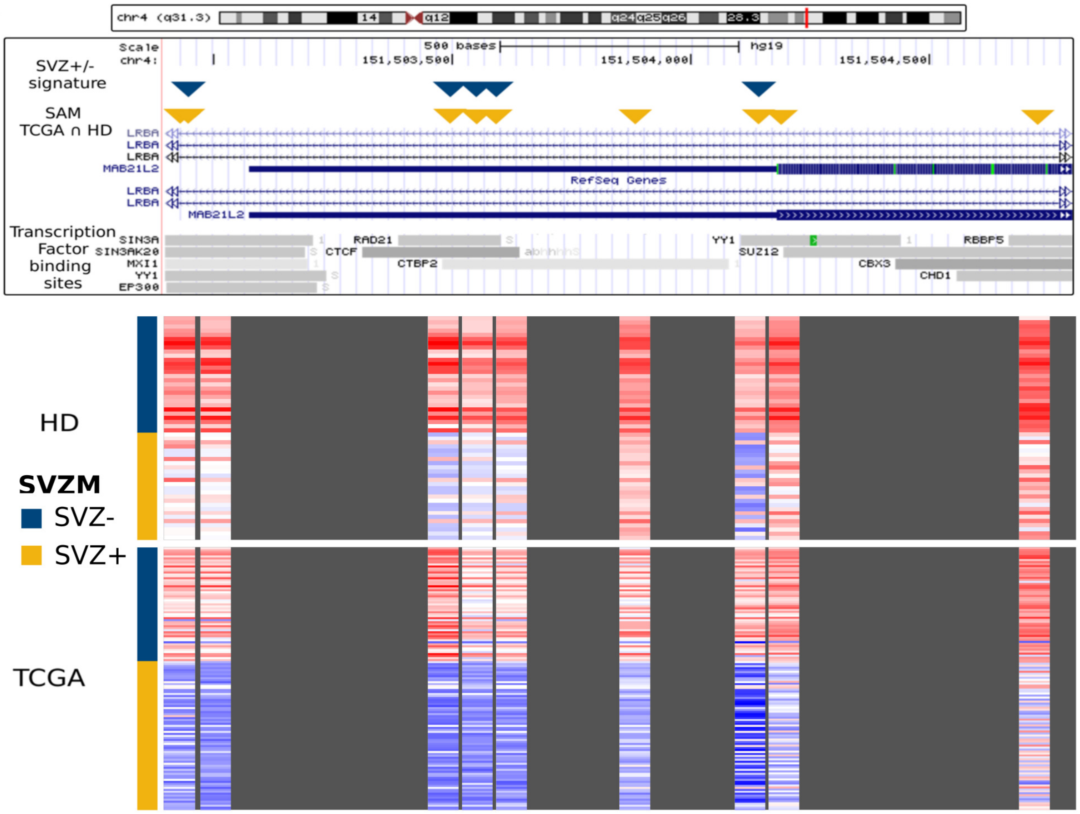

Supplementary Figure 5: Differential methylation of SVZ tumors. A: Differentially regulated CpGs (SAM, 500 permutations, FDR < 0.05). B: Intersect between 15CpG RF signature and 439 differential CpGs. C: Most frequently identified genes by CpG annotation of the 439 intersect CpG set. C: Detailed visualization of the most prominently altered genomic locus on chromosome 4 which is highly abundant in the SVZM signature.

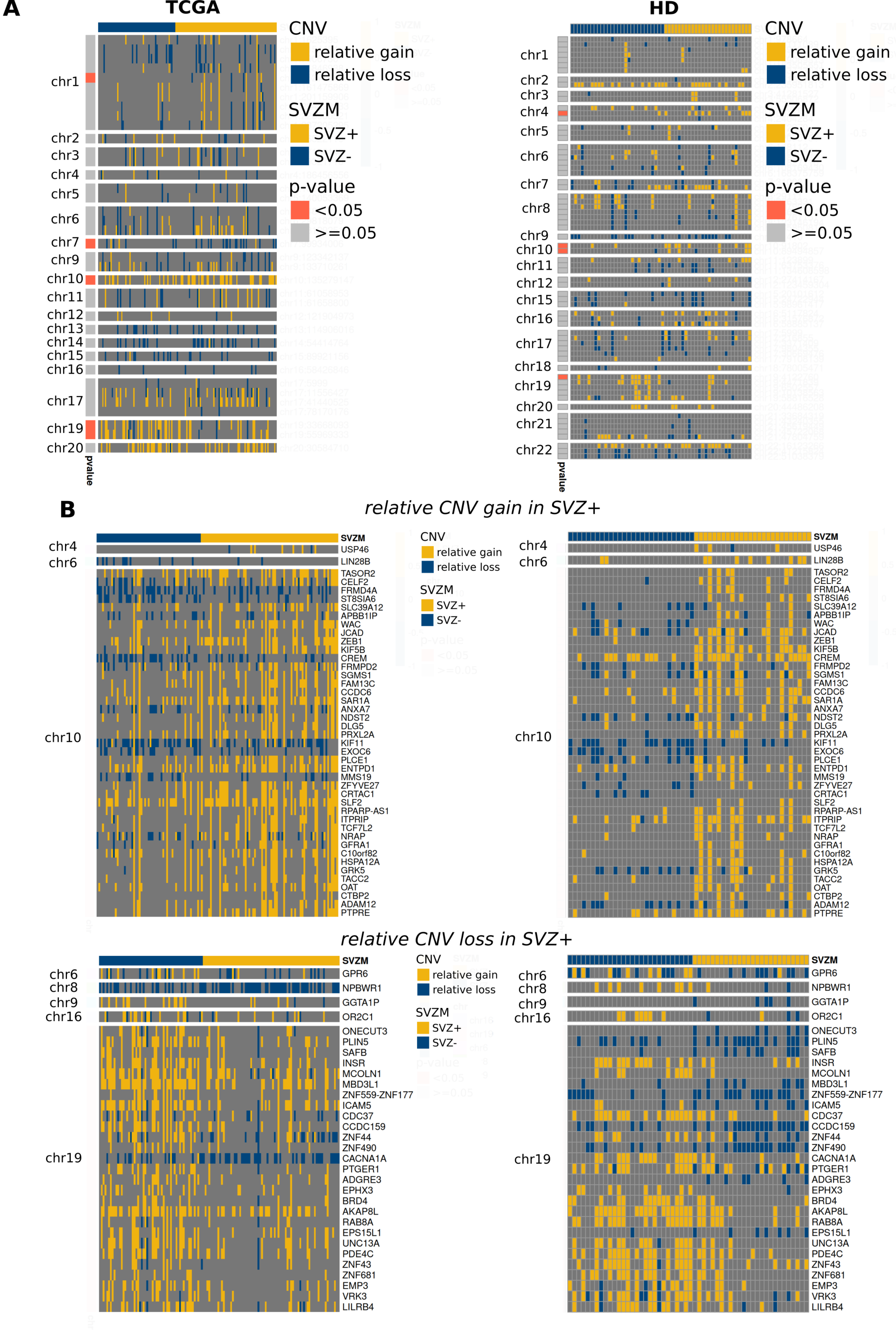

Supplementary Figure 6: CNV alterations on segment (A, all) and gene level, p< 0.05 (B, consensus). Fisher test.

TCGA

A

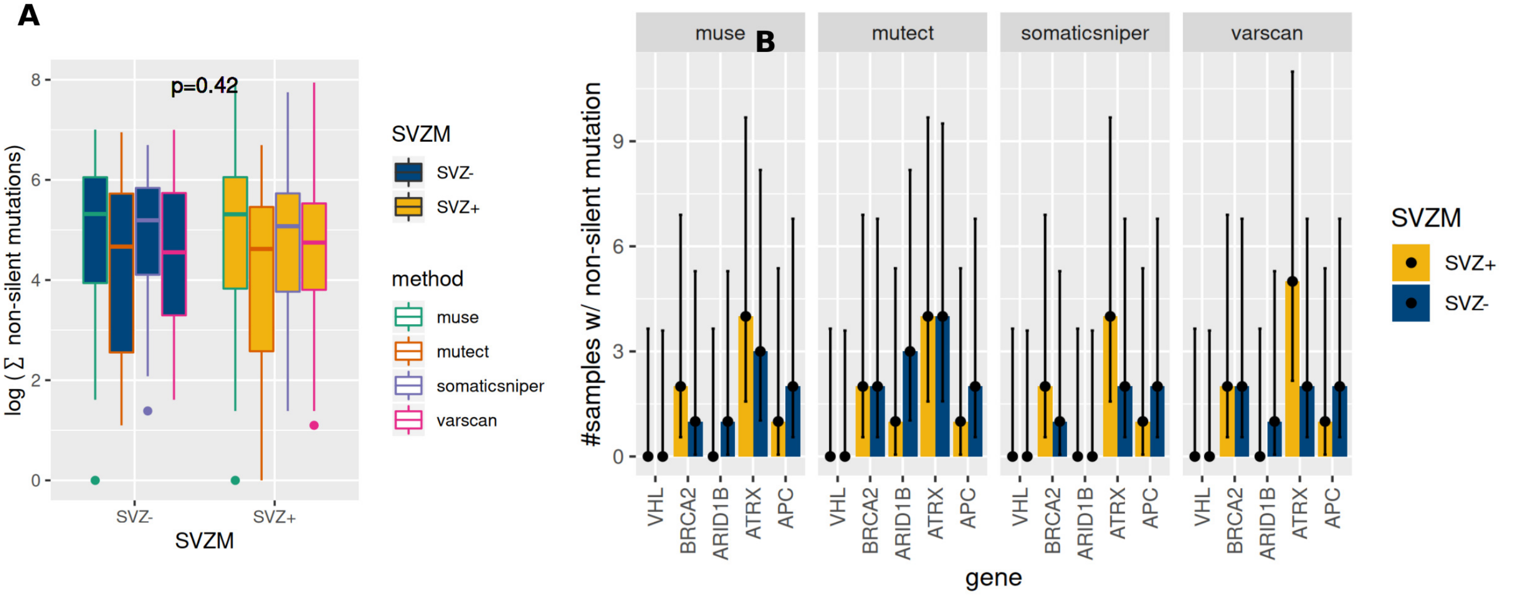

C **Mutational cancer driver genes, IntOGen**

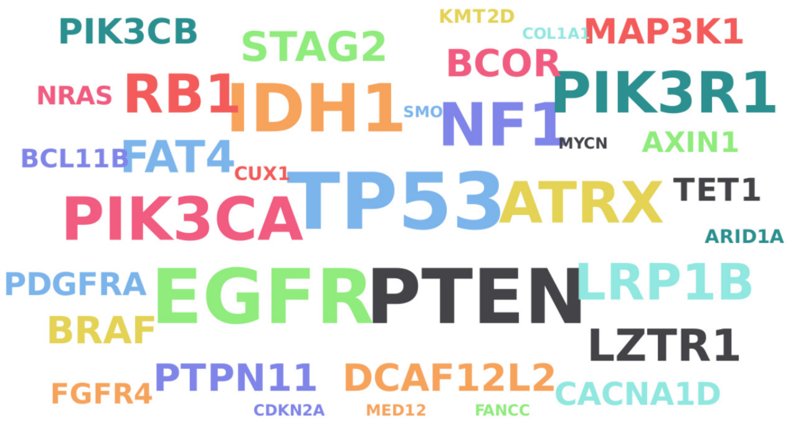

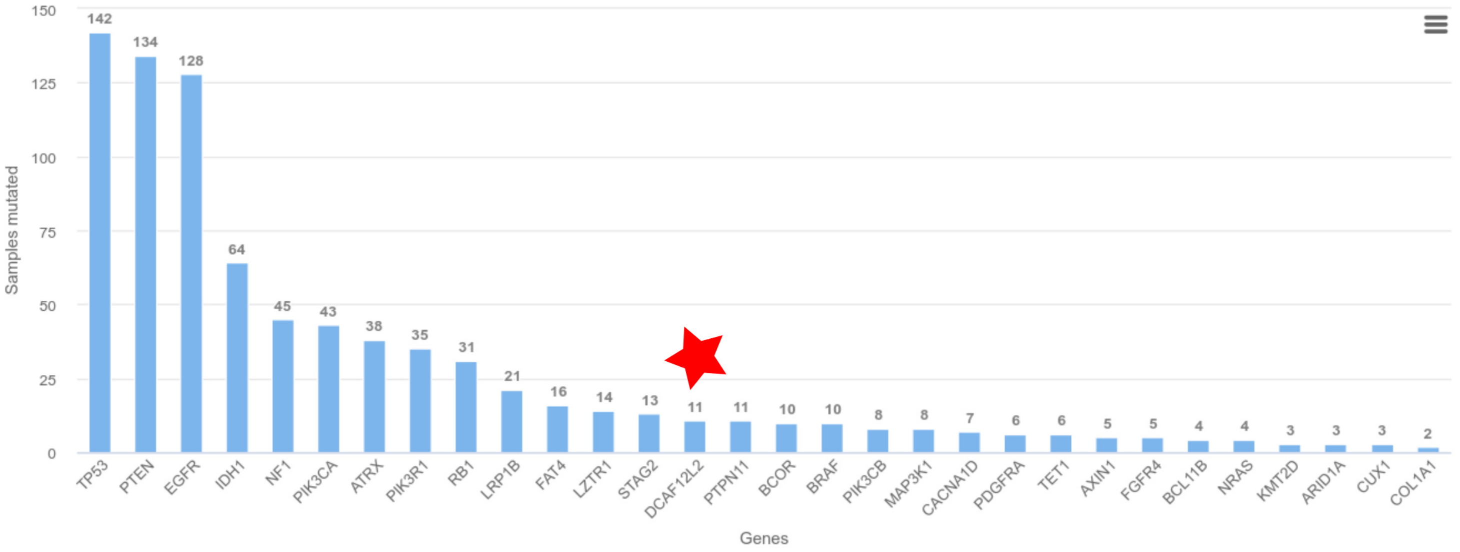

Supplementary Figure 7: Mutations. A: Total numbers of non-silent mutations. B: Selected genes, with Wilson 95% confidence intervals. C: Glioblastoma driver mutations, IntOGen, access date: 2022-04-14. Font size correcponds to number of samples with mutations. Numbers of mutated samples / gene are shown below. Star marks DCAF12L2, identified as being differentially mutated.

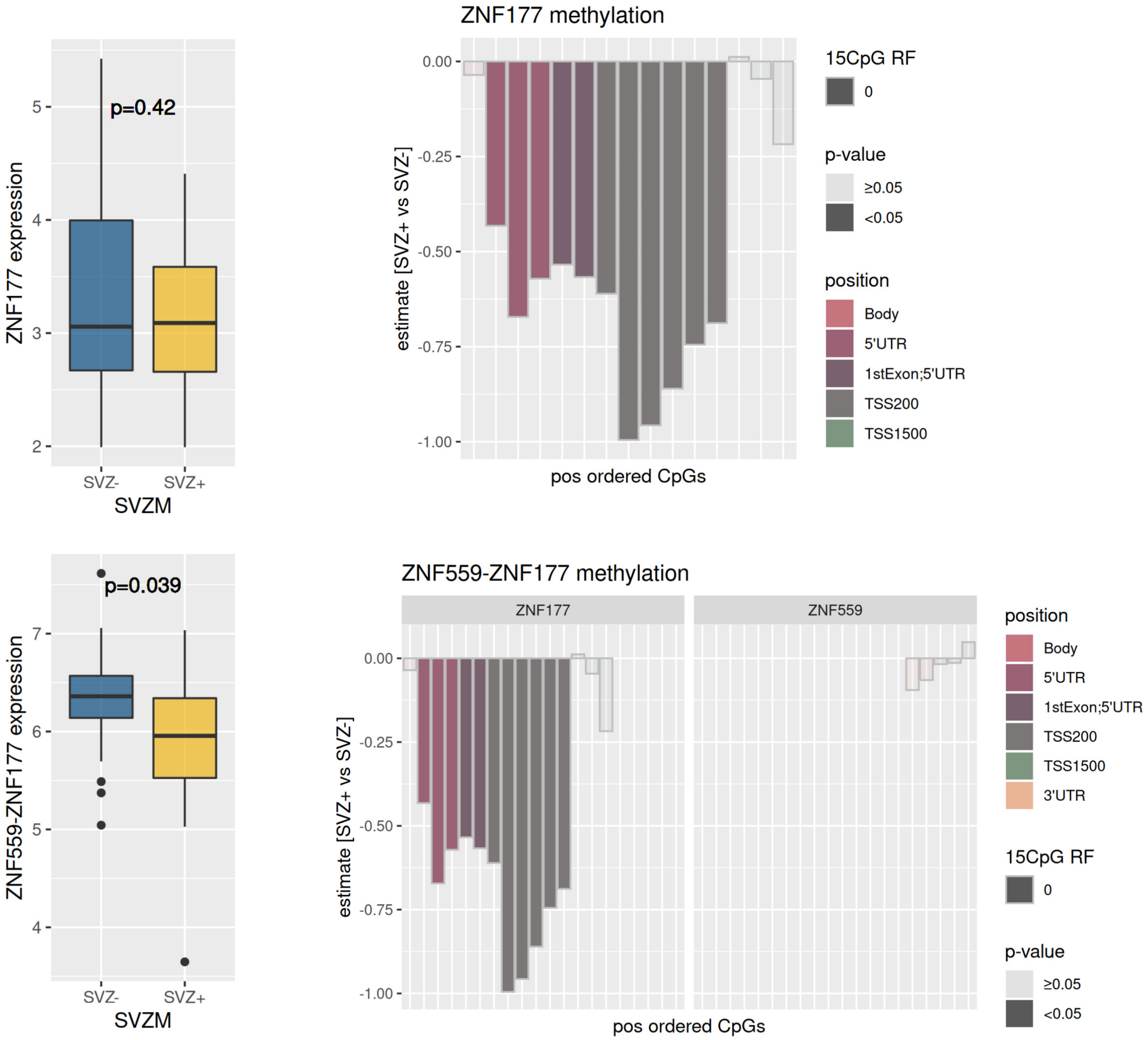

Supplementary Figure 8

Nine ZNF177 CpGs were identified in consensus CpGs, ZNF599-ZNF177 alterations were detected on expression level and copy number level (loss). Our finding is in line with previous literature suggesting an important role ZNF599-ZNF177. It is among the top 10 oncoscore genes (07/2021) and differentially methylated/regulated e.g. in lung, head and neck and colorectal cancer [1-4].

1. Diaz-Lagares, A., et al., *A Novel Epigenetic Signature for Early Diagnosis in Lung Cancer*. Clin Cancer Res, 2016. 22(13):p. 3361-71.
2. Lleras, R.A., et al., *Hypermethylation of a cluster of Kruppel-type zinc finger protein genes on chromosome 19q13 in oropharyngeal squamous cell carcinoma*. Am J Pathol, 2011. 178(5): p1965-74.
3. Chung, H.H., et al., *A Novel Prognostic DNA Methylation Panel for Colorectal Cancer*. Int J Mol Sci, 2019. 20(19).
4. Piazza, R. et al., *OncoScore: a novel, Internet-based tool to assess the oncogenic potential of genes*. Sci Rep, 2017. 7:. 46290.

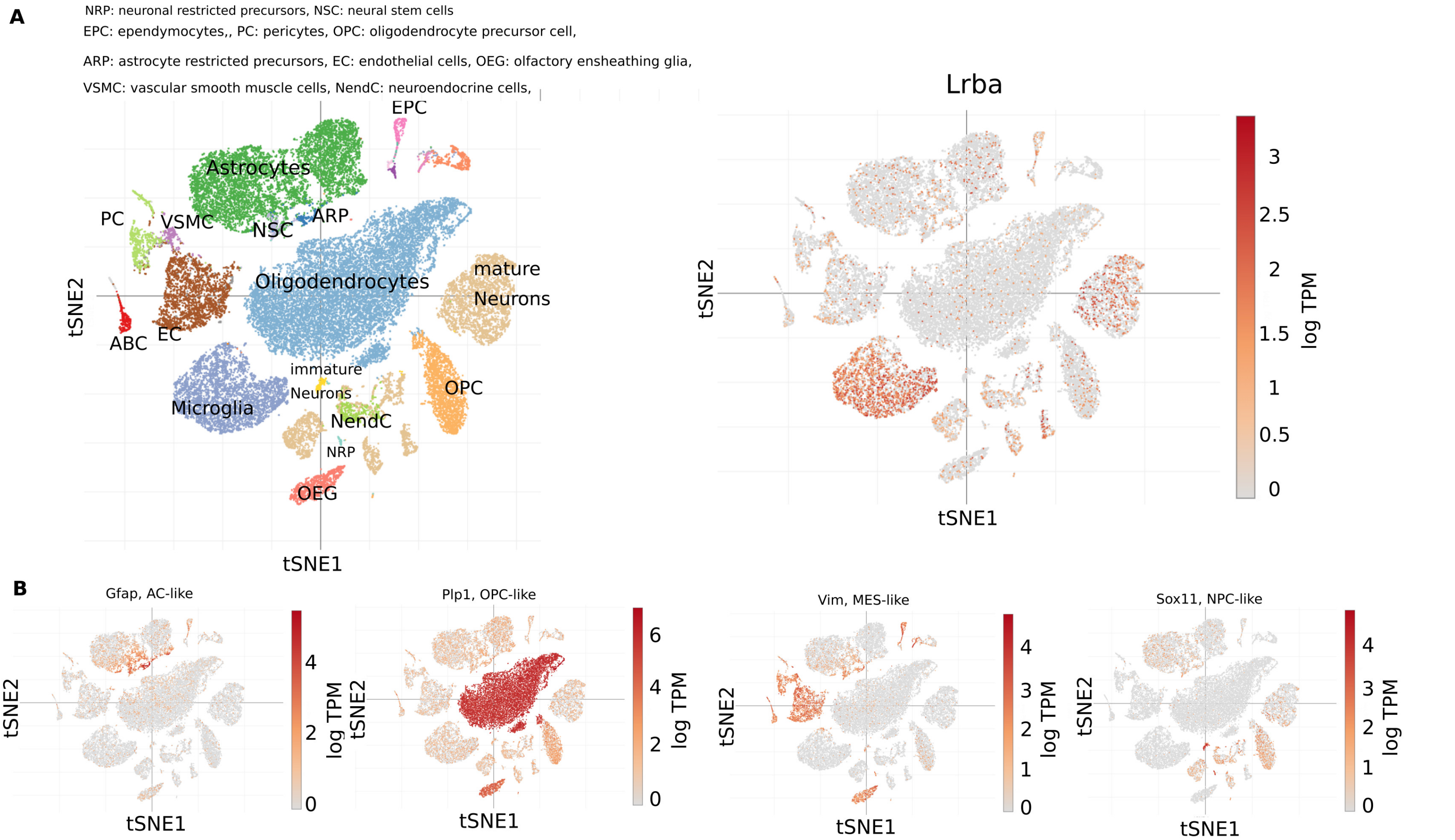

Supplementary Figure 9: (A) Analysis of single cell RNAseq data (Xierakis et al [2]) confined expression of LRBA mRNA (red/brown coloring of positive cells, right side) to the microglia compartment (dark blue cluster, left side) in healthy mouse brains.
B) In analogy, cellular distribution of key biomarker for identification of GBM subtypes as reported by Neftel et al. [1] was also allocated in this scRNAseq data, i.e. Vimentin (Vim) expression for the mesenchymal subtype (MES-like) was confined to endothelial cell (EC), vascular smooth muscle cells (VSMC) and olfactory ensheating glia (OEG), Sox11 (NPC-like) expression was most frequently detected in neuronal restricted precursor cells (NRP) and immature neurons, to a lesser degree in oligodendrocyte precursor cells (OPC) and mature neurons, Plp1 (OPC-like) was abundalty expressed in Oligodendrocytes, OEG and a fraction of OPC, Gfap (AC-like) was expressed in a subset of Astrocytes, neural stem cells (NSC) and astrocyte-restricted precursors (ARP).

[1] Neftel, C., Laffy, J., Filbin, M.G., Hara, T., Shore, M.E., Rahme, G.J., et al. (2019). An Integrative Model of Cellular States, Plasticity, and Genetics for Glioblastoma. Cell 178(4), 835-849 e821. doi: 10.1016/j.cell.2019.06.024.
[2] Ximerakis, M., Lipnick, S.L., Innes, B.T., Simmons, S.K., Adiconis, X., Dionne, D., et al. (2019). Single-cell transcriptomic profiling of the aging mouse brain. Nat Neurosci 22(10), 1696-1708. doi: 10.1038/s41593-019-0491-3.

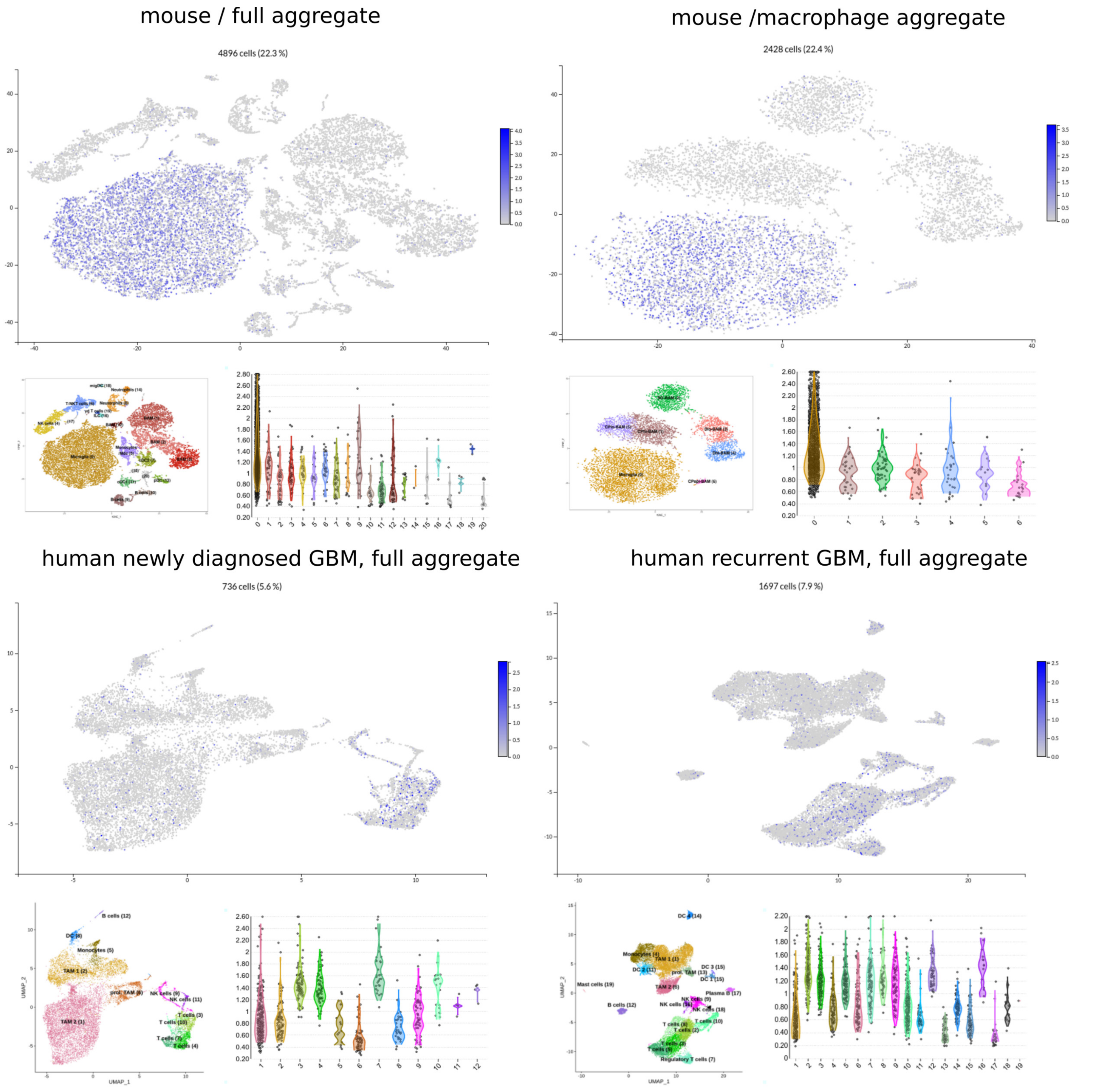

Supplementary Figure 10: Distribution of LRBA expression in mouse and human single cell glioblastoma data from the Brain Immune Atlas (https://www.brainimmuneatlas.org/, [1]). Mouse datasets show high Lrba expression in Microglia, human samples mostly in T-cells. Data accessed on 2022-04-28.

[1] Hannah Van Hove, Liesbet Martens, Isabelle Scheyltjens, Karen De Vlaminck, Ana Rita Pombo Antunes, Sofie De Prijck, Niels Vandamme, Sebastiaan De Schepper, Gert Van Isterdael, Charlotte L. Scott, Jeroen Aerts, Geert Berx, Guy E. Boeckxstaens, Roosmarijn E. Vandenbroucke, Lars Vereecke, Diederik Moechars, Martin Guilliams, Jo A. Van Ginderachter, Yvan Saeys and Kiavash Movahedi. A single-cell atlas of mouse brain macrophages reveals unique transcriptional identities shaped by ontogeny and tissue environment. Nature Neuroscience 2019; 22:1021-35.

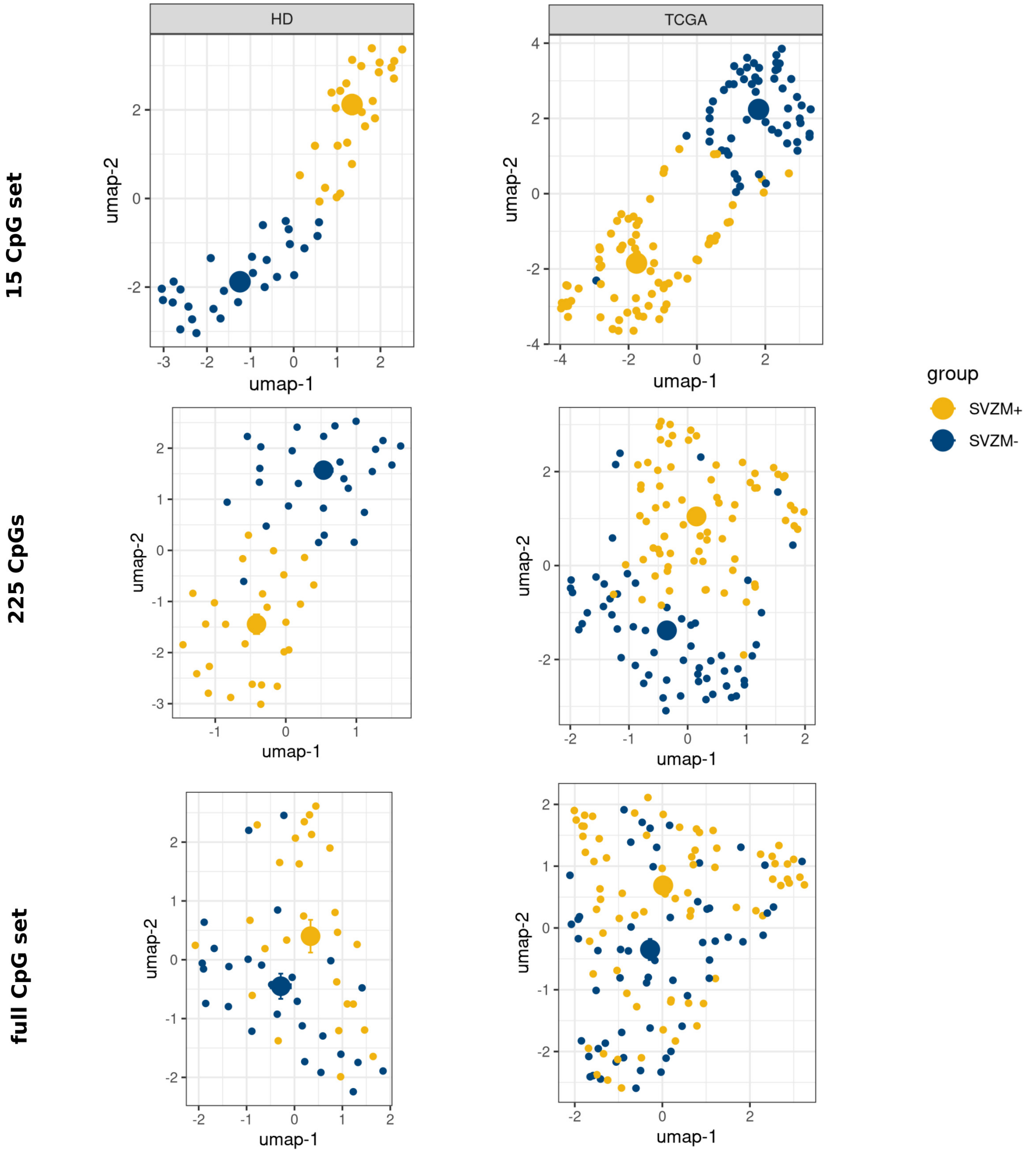

Supplementary Figure 11:

UMAP representation of methylation data with SVZM+ assignment and group centroids (median), showing a separation of SVZM+/- tumors.

Left column: HD data, right: TCGA data.

Upper row: 15 CpG set, middle row: HD 225 CpGs (see Suppl-Fig. 1), 214 CpGs were present in TCGA, bottom row: HD 321584 CpGs, TCGA: 305573 CpGs.

HD: Filtered for X,Y chr, repetetive sequences and SNPs.

TCGA: Intersect with probes present in HD data.

M-values.

50	Odds Ratio	95% CI	p-value
SVZM			
SVZ+	3.37	1.22-9.34	**0.019**
RCHT			
yes	0.07	0.02-0.20	**<0.001**
MGMT			
unmethylated	2.85	1.01-8.02	**0.048**

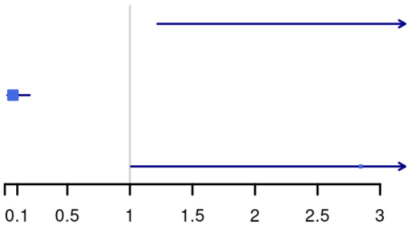

50	Odds Ratio	95% CI	p-value
MRI			
SVZ-	0.44	0.16-1.23	0.117
RCHT			
yes	0.06	0.02-0.20	**<0.001**
MGMT			
unmethylated	2.97	1.05-8.40	**0.041**

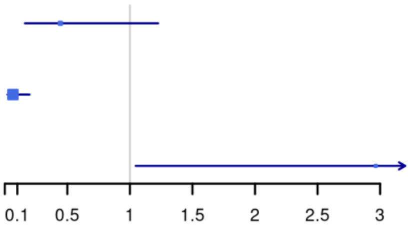

Supplementary Figure 12: Multivariate survival analysis using a parametric model with loglogistic distribution. RCHT: Radio-Chemotherapy.

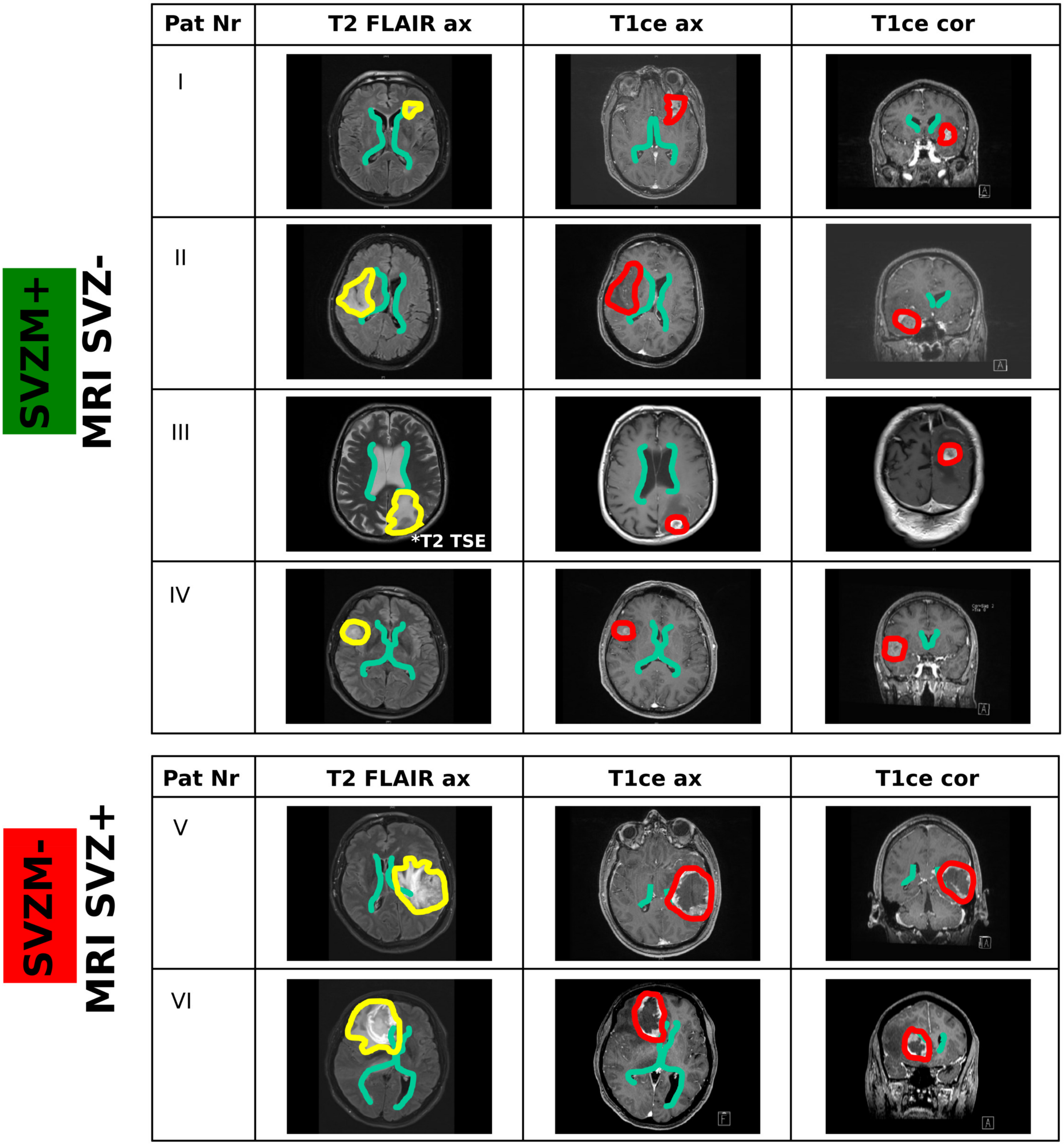

Supplementary Figure 13: Cases with discordant MRI / methylome SVZ classification, see also Figure 2. Tumor extension on FLAIR sequence is marked in yellow, on T1 in red. Relevant ventricular borders are highlighted in green. A careful look at all dimensions (axial ax., coronal cor.) and sequences (T2 FLAIR and T1 contrast enhanced, T1ce) identified ventricle zone proximity in SVZM+ classified tumors. Tumor association with ventricular zone is also visible in MRI-SVZ+ but SVZM- tumor classfication underscores the challenge to discriminate primary association vs. secondary invasion to this zone solely by imaging principles.

Clinical implications of a well-established classifier could be, besides prognosis prediction, a potential use in target volume delineation and inclusion of fibertracks along the SVZ. Furthermore, intentionally inclusion of parts of the SVZ in the target volume could be discussed. Treatment escalations in systemic therapies, RT and potentially tumro treating fields for SVZM+ patientes are meritedbased on the inferior prognosis. A future perspective could be to train machine learning algorithms to predict SVZM allocation.

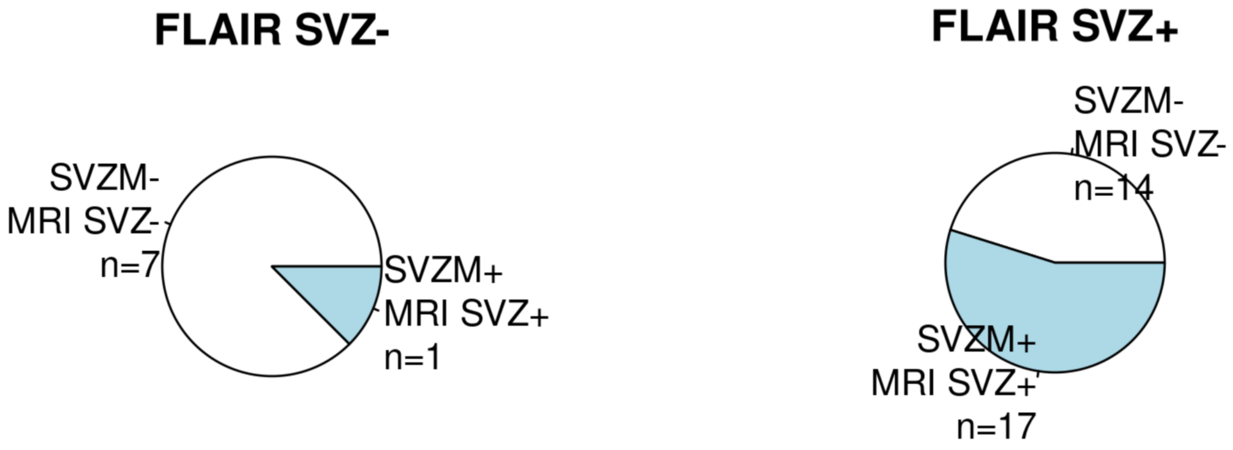

FLAIR SVZ- -> high probability for T1ce SVZ-

FLAIR SVZ+ -> T1ce SVZ+ or SVZ-

Supplementary Figure 14: Concordance of T2FLAIR and T1ce tumor localization assignment for tumors with equal T1ce MRI and SVZM classification for which T2 FLAIR sequences were available (n=39).
By FLAIR sequence as SVZ- classified tumors were in all but one case also T1ce SVZ- (left).
FLAIR SVZ+ classfied tumors were nearly equally classified as SVZ+/- on T1ce sequence (right).

Feature	n	%
**All**	47	100
**Sex**		
female	21	44.680
male	25	53.190
**Age**		
	64.55	97.870
	[23.38;85.66]	
**RT**		
yes	12	25.530
**TotalDose**		
	60	19.150
	[32.7;160]	
**TMZ**		
yes	11	23.400
**TMZadjuvant**		
yes	9	19.150

Feature	n	%
**All**	132	100
**Sex**		
female	55	41.670
male	75	56.820
**Age**		
	61.94	98.480
	[21.8;85.66]	
**RT**		
yes	39	29.550
**TotalDose**		
	60	24.240
	[8;160]	
**TMZ**		
yes	36	27.270
**TMZadjuvant**		
yes	32	24.240

Feature	n	%
**All**	39	100
**Sex**		
female	17	43.590
male	22	56.410
**Age**		
	61.35	100
	[44.18;85.66]	
**RT**		
yes	31	79.490
**TotalDose**		
	60	64.100
	[8;160]	
**TMZ**		
yes	30	76.920
**TMZadjuvant**		
yes	27	69.230

Supplementary Table 1: Patient characteristics of the TCGA-GBM data. Median and ranges in brackets for age and RT total dose [Gy].
