# Supplementary figures and images for: DNA-methylome-assisted classification of patients with poor prognostic subventricular zone associated IDH-wildtype glioblastoma
Source: Acta Neuropathol. 2022 Jun 4;144(1):129–42. doi: 10.1007/s00401-022-02443-2 (PMC9217840; doi:10.1007/s00401-022-02443-2)

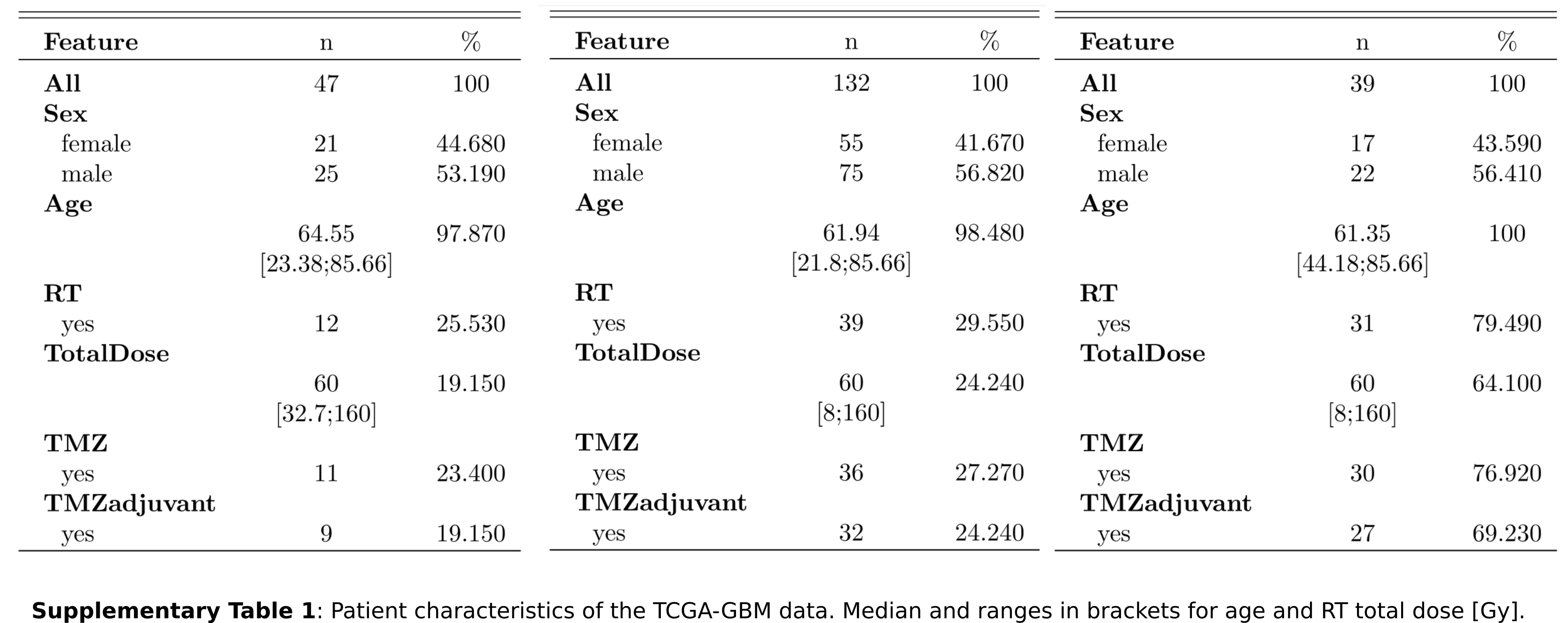

Supplement: Supplementary file 2 — Supplementary file2 (TIFF 611 kb) [file 401_2022_2443_MOESM2_ESM.tiff]
